# Jupyter and Galaxy: Easing entry barriers into complex data analyses for biomedical researchers

**DOI:** 10.1371/journal.pcbi.1005425

**Published:** 2017-05-25

**Authors:** Björn A. Grüning, Eric Rasche, Boris Rebolledo-Jaramillo, Carl Eberhard, Torsten Houwaart, John Chilton, Nate Coraor, Rolf Backofen, James Taylor, Anton Nekrutenko

**Affiliations:** 1 Bioinformatics Group, Department of Computer Science, Albert-Ludwigs-University, Freiburg, Freiburg, Germany; 2 Center for Biological Systems Analysis (ZBSA), University of Freiburg, Freiburg, Germany; 3 Department of Biochemistry and Biophysics, Texas A&M University, College Station, Texas, United States of America; 4 Centro de Genética y Genómica, Universidad del Desarrollo, Santiago, Chile; 5 Department of Biology, Johns Hopkins University, Baltimore, Maryland, United States of America; 6 Department of Biochemistry and Molecular Biology, The Pennsylvania State University, University Park, Pennsylvania, United States of America; Ontario Institute for Cancer Research, CANADA

## Abstract

What does it take to convert a heap of sequencing data into a publishable result? First, common tools are employed to reduce primary data (sequencing reads) to a form suitable for further analyses (i.e., the list of variable sites). The subsequent exploratory stage is much more ad hoc and requires the development of custom scripts and pipelines, making it problematic for biomedical researchers. Here, we describe a hybrid platform combining common analysis pathways with the ability to explore data interactively. It aims to fully encompass and simplify the "raw data-to-publication" pathway and make it reproducible.

## Author summary

Galaxy users can utilize a large number of tools and workflows. What they could not previously do is run ad hoc scripts and arbitrary tools within their Galaxy instance. This was very limiting, as initial analyses of data often involve interactive exploration with tools like Jupyter or RStudio—powerful platforms that are becoming increasingly popular in life sciences. Here, we showcase Galaxy Interactive Environment framework, designed to combine Galaxy's tools and workflows with environments such as Jupyter.

## Introduction

Trees, rivers, and the analysis of next generation sequencing (NGS) data are examples of branching systems so ubiquitous in nature [1]. Indeed, numerous types of NGS applications (i.e., variation detection, analyses of DNA/Protein interactions [ChIP-seq] or transcriptome [RNA-seq]) share the same initial processing steps (quality control, read manipulation and filtering, mapping, post-mapping thresholding, etc.), making up the trunk and main branches of this tree. Each of these main branches subsequently gives off smaller offshoots (variant calling, RNA-seq, ChIP-seq, and other "seqs") that, in turn, split further as analyses become focused towards the specific goals of an experiment. As we traverse the tree, the set of established analysis tools becomes increasingly sparse, and it is up to an individual researcher to come up with statistical and visualization approaches necessary to reach the leaves (or fruits) that represent conclusive, publishable results. Consider transcriptome analysis as an example. Initial steps of RNA-seq analysis (in our tree analogy, these are trunk and main branches), such as quality control, read mapping, and transcript assembly and quantification are reasonably well established. Yet completion of these steps does not produce a publishable result. Instead, there is still the need for additional analyses (progressively smaller branches of our tree), ranging from simple format conversion to statistical tests and visualizations. Thus, every NGS analysis can, in principle, be divided into two stages. The first stage involves processing of raw data using a small set of common, generic tools. This stage can be scripted and automated and also lends itself to building graphical user interfaces (GUIs). The second stage involves a much greater variety of tools that need to be customized for every given experiment (in many cases, there are no tools at all, and custom scripts need to be developed). As a result, it is not readily coerced into a handful of automated routines or generic GUIs.

The main motivation for this work was the development of a system wherein biomedical researchers can perform both stages of data analysis: initial steps using established tools and exploratory and data interpretation steps with ad hoc approaches. Merging both steps into a unifying platform will lower entry barriers for individuals interested in data analysis, significantly improve reproducibility of published results, ease collaborations, and enable straightforward dissemination of best analysis practices.

## Materials and methods

Jupyter integration into Galaxy takes advantage of the recently developed and increasingly popular Docker containerization platform (https://www.docker.com). It uses the Interactive Environment (IE) plug-in functionality written for Galaxy that also allows integration of other similar tools such as RStudio. It consists of an Interactive Environment Entry Point (IEEP) and an associated configuration file. The IE configuration allows administrators to set it so that all data transfer is done via Secure Socket Layer (SSL), which is useful for production instances. Additionally, individual sites can specify custom Docker images instead of the default provided Jupyter notebook, allowing administrators to craft Docker images more specific to their users. The default image will be downloaded and installed from Docker Hub or quay.io—popular Docker image hosting services. The default Docker image is specifically crafted for use in conjunction with the Jupyter Interactive Environment (see below).

The IEEP launches a Docker container on a random port for communication and configures it to access Galaxy through environment variables passed to the container. This container, by the very nature of Docker itself, is isolated from the filesystem and processes on the Galaxy server. In addition, administrators can configure Docker containers to run on remote computing resources using Docker's built-in client/server architecture. Doing so also provides an additional layer of security by fully resource-separating the IE container from the Galaxy server. For greater scalability, Docker Swarm, the distributed Docker container engine provided with the Docker software, is supported. The host and port on which the container is running are stored in a database on the Galaxy server so that Galaxy and the Dockerized Jupyter web service can communicate securely while isolated from the rest of the Galaxy instance for security reasons.

The container is built on top of the official “jupyter/minimal-notebook” image (which is maintained by Project Jupyter) and provides a Jupyter server, along with its dependencies, such as NumPy [2], SciPy [3], and Matplotlib [4]. Additionally, the image contains several Jupyter kernels (different programming language environments), such as R, Ruby, Haskell, Julia, and Octave. By utilizing a Docker image with a full suite of scientific analysis tools and libraries, users are able to immediately perform their analysis and calculations. In the Python kernel, additional packages can be installed with the python package manager called “pip.” The same is true for the other kernels and their associated package managers. Moreover, tools that can be installed and run in a nonprivileged user account can be added to the container on demand. Once the container has launched on the backend, it is embedded inside the Galaxy interface, at which point it can be used to interactively program, develop, and analyze data in any of the aforementioned programming languages. Each invocation of the IE by a Galaxy user results in the launch of a new Docker container, meaning that users are isolated from each other. If the page with the Interactive Environment is closed by the user, Galaxy instructs Docker to terminate the process. Additionally, during Docker container startup, a service is launched that monitors whether the IE is still being used by checking the network traffic so that it can automatically terminate itself when the IE is no longer in use.

Within the Jupyter notebook, two important custom functions are defined that enable the user to load data from the history or store data to the Galaxy history using the Galaxy API [5]. The “get” function expects one parameter: the numerical identifier of the dataset as shown in the history. The retrieved dataset is stored as a file inside the container, which can be accessed via the usual means for the language kernel in use (e.g., the “open” function). The “put” function automatically builds a connection to the host Galaxy instance and transfers a specified file from inside the Docker container to the user's history. Thus, any dataset the user has access to in Galaxy can be loaded into the notebook, datasets can be combined or modified programmatically, and the results can be written back to the history. The entirety of Galaxy–IE communication occurs between the Galaxy host and the Docker container, without the need for the user to upload or download data to their personal workstation. This is not only faster in most cases, but it also has positive implications on data security, as the data did not leave the compute center.

In addition to the SSL-secured dataset transfer already mentioned, all of Docker's security and resource control features are available to the administrator. These include CPU and memory limits and SSL-secured client/server communication. Additionally, every container can be password protected if desired—a password is randomly generated and presented to the user during startup of the container in his/her web browser. Notebooks can be saved to the Galaxy history at any time; once in Galaxy's history, they can be inspected like any other Galaxy dataset, allowing for a read-only view of the analysis steps that we run. Additionally, notebooks can be reused. A new Jupyter instance is created that retains the stored work. This functionality ensures the reproducibility of data analysis and is therefore an essential feature of the Jupyter Interactive Environment.

## Results/Discussion

To avoid "reinventing the wheel" in designing our platform, we first evaluated existing systems that can be leveraged to fulfill our goals. For the first stage of the analysis, we needed a system that exposes existing common tools through a unifying interface and makes computational infrastructure needed to perform large-scale analyses transparent to the user. There are several systems potentially satisfying these requirements, including Genepattern [6], Mobyle [7], CyVerse [8], Galaxy [9], and GenomeSpace [10]. These systems allows users to utilize a large number of tools and workflows, as well as record provenance, ensuring reproducibility of analyses. However, these systems are only as useful as their set of featured tools and do not aid in ad hoc data exploration.

The number of available choices for the second stage of the analysis (ad hoc exploration) is enormous. One can simply use scripting languages, relational databases, spreadsheet applications, or commercial packages to conduct data interpretation. One important feature we sought is the ability for a system to record analysis steps in order to make the final outcome reproducible. In this regard, two well-established open environments designed specifically for reproducible data exploration stood out as de facto standards in scientific computing: IPython/Jupyter [11] and RStudio [12].

In the end, we proceeded with Galaxy (due to its considerable user base [http://bit.ly/gxyStats]) as the underlying platform for management of data, tools, and infrastructure and Jupyter as an initial data exploration plug-in (at the time of writing, RStudio has also been integrated and is being tested). Galaxy is a web-based analysis environment that exposes tools using GUI, allows combining them into workflows, and is supported by software and hardware infrastructure suitable for analysis of very large multisample datasets. The benefit of Galaxy is that anyone with a web browser can perform analyses in a straightforward manner without being concerned with how or where the underlying software is executed. In addition, Galaxy is used in other scientific domains distinct from life sciences (e.g., [13]), and thus, the approach described here will benefit other disciplines as well. Jupyter (formerly known as IPython) is an interactive programming environment allowing reproducible data analysis with over 60 programming languages (such as Python, Julia, R, and others). It is built around the concept of Jupyter notebook—a web application allowing the combining of executable programming language code with visualization and explanatory annotations into a single "live" document. The advantage of Jupyter is that there is essentially no limit on what one can do, as supported languages and underlying libraries enable the full spectrum of data analyses. However, to be useful, Jupyter requires programming and data management skills, as well as access to computational infrastructure. Table 1 contrasts the pros and cons of the two platforms (Galaxy and Jupyter) and shows that their combination provides an almost perfect analysis solution for biomedical domain.

**Table 1:** Congruence between Galaxy and Jupyter as a function of their pros and cons.

Feature	Galaxy	Jupyter	Galaxy/Jupyter
Low barrier of entry for a naive user	*		*
Versatility of available tools		*	*
Provenance tracking	*	*	*
Hardware backend for processing of large datasets	*		*
Attractive to experimentalists	*		*
Attractive to bioinformaticians and data scientists		*	*

**Fig 1:**
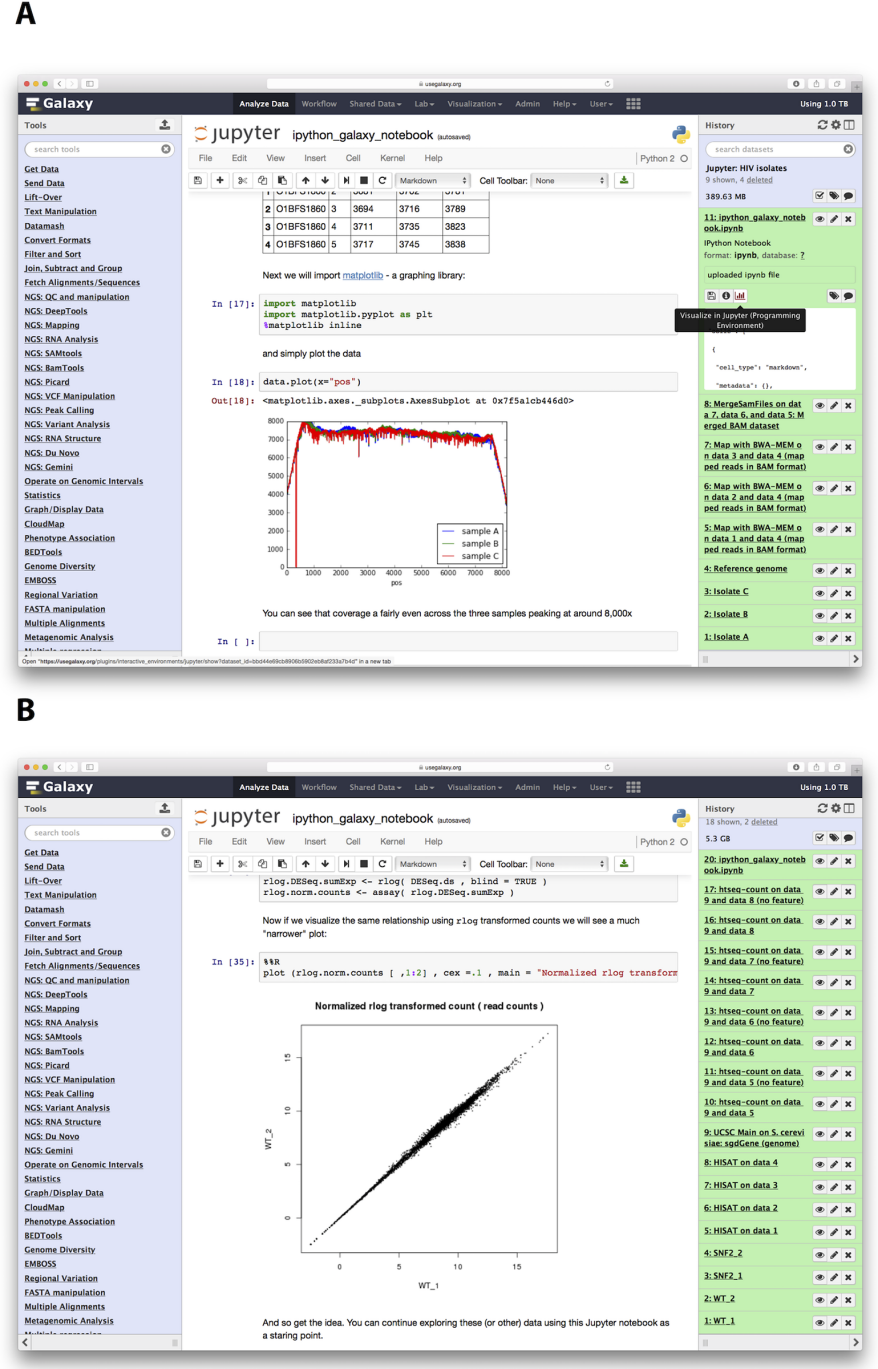
Overview of steps involved in performing analyses outlined in Examples 1 and 2. **A**. Example 1. Right (green) side of Galaxy interface is the history pane. The analysis begins with uploading three Illumina datasets (datasets 1–3) and a reference genome sequence (dataset 4). Datasets are mapped to the reference genome with bwa-mem (datasets 5–7) and read groups are assigned (datasets 8–10). This allows resulting BAM datasets to be merged into a single BAM file (dataset 11). At this point, the Jupyter IE is launched. Lower part of the notebook is visible in the center pane, showing the read coverage distribution for the three isolates (three different colors). **B**. A similar screenshot for Example 2. Here, Illumina reads for two RNA-seq replicates from wildtype and snf2 knock-out are mapped against the *Drosophila melanogaster* genome (dm3) using HiSat split mapper. Next, HTSeq-count takes BAM datasets generated by HiSat and, using gene annotation for dm3 genome downloaded from the UCSC Table Browser (history dataset 9), computes per-gene read counts. These counts are then imported to Jupyter (center pane) to perform normalization and variance shrinkage calculations using Bioconductor's DESeq2 package.

How can these two very dissimilar applications, Galaxy and Jupyter, work together in practice? In Galaxy, datasets corresponding to each step of analysis are recorded in the history as "history items" (right panes of the interface in Fig 1). Once an analysis reaches the point at which there are no tools available for the next step, it is time to switch to Jupyter. This is done by clicking a button adjacent to the dataset, which will start an isolated instance of Jupyter (or other so-called Interactive Environments, such as RStudio) directly within the Galaxy interface. This instance interacts with Galaxy's Application Programming Interface (API) using custom methods for transferring data back and forth from Galaxy's history. Jupyter's "notebooks" allow ad hoc analyses to be recorded automatically, providing the utmost level of reproducibility during data exploration. After performing analyses, the user can save the notebook as a Galaxy history item that can be downloaded and used as a template for a new tool. It can be re-run with changed parameters on different datasets, and like any other history item, it can be shared with other Galaxy users. Additionally, in the future, it will be possible to export the code, aiding in rapid development of Galaxy tools. It can also be converted into a PDF, documenting details of the analyses. Here, we used this functionality to generate S1, S2 and S3 Files, corresponding to the three examples described below.

To demonstrate the utility of Galaxy/Jupyter integration, we devised three examples. In all three cases, we break down the analysis in parts 1 (Galaxy) and 2 (Jupyter). For each example, part 1 involves processing and mapping of the sequenced reads. In the first example, we use a simple combination of command line tools and Python scripting language to plot read coverage across the HIV genome. In the second example, we leverage Python and R to normalize read counts and shrink variance in an RNA-seq experiment. Finally, in the most complex example, we perform data processing and replicate main summary figures from our previous study [14]. This third example demonstrates the capabilities of Galaxy and Jupyter to process large, multisample datasets. In this example, Galaxy's power is leveraged for mapping and processing of hundreds of datasets, and Jupyter is used for the final interpretation and replication of published figures. These examples can be viewed in S1–S3 Files, or they can be interacted with in a live Galaxy/Jupyter instance by using the links indicated below (see S1 Fig for an explanation of how to use these links).

### Example 1: Building a genome coverage plot

HIV-1 was resequenced from the blood of a single individual across three time points with the ultimate goal of tracking nucleotide substitutions of the viral genome through time (simulated reads were generated for this example). After assessing the quality of the reads and mapping against the HIV-1 genome with bwa [15] within Galaxy, we wanted to visualize read coverage across each sample to decide if further analyses are warranted. However, the main public Galaxy server did not have a dedicated tool for this purpose. Normally, the analysis will stop at this point, and only by downloading data and analyzing them offline can one produce the coverage distribution graph needed in this case. Integration of Jupyter to Galaxy changes this. Fig 1 highlights each step of this analysis, resulting in the coverage distribution graph. The entire analysis can be seen in the Galaxy history, accessible at http://bit.ly/ie-hiv (see S1 File).

### Example 2: Normalizing read counts for an RNA-seq experiment

In this example, we use a subset of RNA-seq data from a dataset published by Schurch et al. [16] (SRA accession ERP004763) consisting of 48 replicates of two *Saccharomyces cerevisiae* populations: wildtype and snf2 knock-out mutants. For simplicity, we selected only two replicates for each wildtype and snf2 knock-outs. Here, we first use Galaxy's existing RNA-seq tools to map reads against the yeast genome using HiSat [17] and to compute the number of reads per gene region using HTseq-count [18] (Fig 1B; see Galaxy history at http://bit.ly/rnaseq-jupyter and S1 Fig). Datasets are then imported into Jupyter's environment (cells 4–9; see S2 File), where we first merge datasets into a single table by joining them on gene names using Python's Pandas library (cells 10–12). We then proceeded to normalize the counts with DESeq2 [19] (cells 13–30) and assessed the effects of normalization and variance shrinkage on the data (cells 31–35; also see center pane of Fig 1B).

### Example 3. Estimating mitochondrial bottleneck in humans

**Fig 2:**
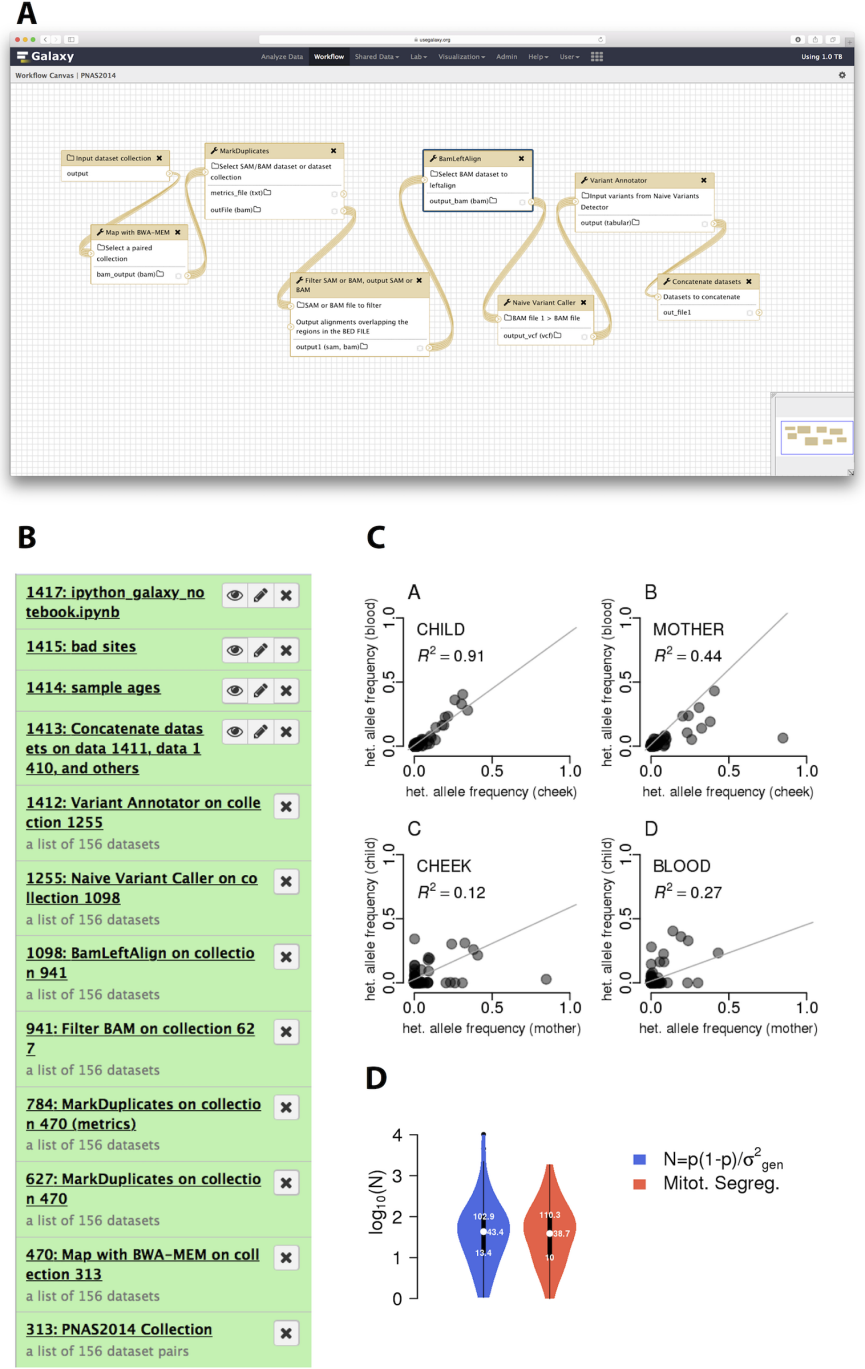
Reanalysis of data from [14] using Galaxy and Jupyter. **A**. Workflow used in the analysis. As an input, the workflow takes a collection of paired Illumina datasets and outputs an unfiltered list of variable sites. **B**. Galaxy history showing all steps of these analyses. It only contains 12 steps because we use dataset collections to combine multiple similar datasets into a small number of history entries. This significantly simplifies processing. For example, collection 313 contains all 312 paired-end Illumina datasets generated for this study. This allows us to deal with just one history item instead of 312. The next item in the history is a collection of BAM datasets generated by mapping each read-pair from collection 313 against human genome (hg38) with bwa-mem. These BAM datasets are de-duplicated (collection 627), filtered (by only retaining reads mapping to mitochondrial DNA, with mapping quality of 20 or higher, and mapped in a proper pair; collection 941), realigned to mitigate misalignment around indels or structural variant calls (collection 1098), and used to call variants with Naive Variant Caller [21]. Finally, we use Variant Annotator to process VCF datasets generated by Naive Variant Caller and to create a list of variants (collection 1412) and the concatenation tool to reduce collection 1412 into a single table (dataset 1413). This dataset is used for further processing with Jupyter. **C**. The relationship of minor allele frequencies for heteroplasmic sites between tissues (panels A and B) and individuals (panels C and D). **D**. Estimates for bottleneck size with (red) and without (blue) accounting for mitotic segregation.

In the third example, we replicate the key analyses reported in a study of human mitochondrial heteroplasmy transmission dynamics, previously published by our group [14]. The goal of this study was to detect heteroplasmies (variants within mitochondrial DNA) and to trace their frequency changes across mother–child transmission events using primary sequencing data generated by [14] (mitochondria is transmitted maternally, and heteroplasmy frequencies may change dramatically and unpredictably during the transmission due to a germ-line bottleneck [20]). The first part of the analysis is performed using Galaxy's mapping and variant calling workflow outlined in Fig 2A. The goal of this part is to generate a preliminary list of sequence variants. The input data consist of over 118 GB of sequencing reads corresponding to 312 fastq datasets (SRA accession SRP047378) derived from 156 samples (39 mothers and 39 children, with two tissues analyzed per individual, each tissue generating two fastq datasets for the forward and reverse read sets, together resulted in the 312 original datasets; Fig 2B, dataset 313). Using Galaxy, we combine all 312 datasets into a single entity, a dataset collection, in order to avoid repetitive tasks (see Galaxy history at http://bit.ly/jupyter-mt, S1 Fig and Fig 2B). The workflow maps the reads and performs de-duplication and extensive filtering of resulting BAM datasets, as well as identifies variable sites. The workflow reduces sequencing reads to a 160 MB data matrix with over 2.6 million rows containing variants for all 156 samples. Despite the fact that we have reduced the primary sequence data to a set of variable sites, this dataset hardly resembles an interpretable result. At this point, exploratory analyses must begin. Unfortunately, it is also the point at which users are forced to leave Galaxy, confounding efforts for reproducibility of the analysis. With Galaxy/Jupyter integration, this deficiency can be avoided. The second part of the analysis begins with starting a Jupyter notebook from inside the Galaxy interface. It proceeds through numerous custom data processing steps and statistical analyses outlined in S3 File. Two main conclusions of this analysis are the positive correlation between the age of the mother and the number of heteroplasmic sites, which has potential implications for the higher rate in mitochondrial DNA (mtDNA) diseases in children born to older mothers (Fig 2C), and the very small size of mitochondrial bottleneck (Fig 2D) at only approximately 40 segregating units.

The above three examples highlight the power of combining ad hoc programmatic analyses with a collection of robust tools already provided by Galaxy. In our opinion, this has the potential to streamline the ways in which biomedical data analysis is performed. In particular, we see the following implications:

#### Lowering entry barriers

At this point, it is widely acknowledged that every biomedical researcher should be able to at least try performing basic data manipulation and analysis tasks. In practice, they are often discouraged from doing this by lack of familiarity with systems such as Jupyter or RStudio and may not know how to configure them for initial use. Integration of Jupyter into Galaxy gives these users a risk-free opportunity to try and learn basic exploratory skills without the need to install or maintain anything.

#### Allowing reuse and experimentation

Jupyter notebooks are designed to be shareable, just like Galaxy's workflows, histories, and datasets. This significantly simplifies reuse: one may, for instance, simply import the notebook we developed in Example 3 and apply it to their own data. This also aids in experimentation: what would happen if, in the analysis described by [14], we were to use a different mapper and/or variant caller? It is easy to answer this question by applying the existing notebook to a set of variant calls produced with an alternative workflow.

#### Increasing collaborative possibilities

Galaxy is popular with biologists due to the ability to run complex analyses without the need to use the command line interface (CLI). However, this is also the reason why many computational scientists are skeptical and often avoid the platform: they feel constrained without the ability to have full control over tool execution and workflow construction. Integration of Jupyter will bring the two communities closer: computational scientists and bioinformaticians will be able to develop analyses using interactive environments in the form of notebooks, which will immediately be usable by biomedical researchers.

One potential argument against environments such as Jupyter (particularity in the context of life sciences, in which a majority of users are new to data analysis) is the need for an initial set of programming/scripting skills. This is true: such a need unavoidably exists. However, our approach is allowing users to alternate between the comfort of Galaxy's interface and the versatility of Jupyter. We believe that this gives users the opportunity to experiment with simple programming tasks to gain skills and confidence to explore further. As such, our system (and its subsequent evolution) is the first step in making more and more researchers within the life sciences familiar with scientific computing principles.

## Supporting information

S1 Fig Importing Galaxy's history and starting Jupyter notebook. Go through steps highlighted in this figure to start Jupyter notebooks described in examples 1, 2, and 3.
(PDF)

S1 File Jupyter notebook for Example 1. (PDF)

S2 File Jupyter notebook for Example 2. (PDF)

S3 File Jupyter notebook for Example 3. (PDF)
