# Supplementary material for: Jupyter and Galaxy: Easing entry barriers into complex data analyses for biomedical researchers
Source: PLoS Comput Biol. 2017 May 25;13(5):e1005425. doi: 10.1371/journal.pcbi.1005425 (PMC5444614; doi:10.1371/journal.pcbi.1005425)

A. Create Galaxy account

If you do not have Galaxy account — create one. If you do — log in.

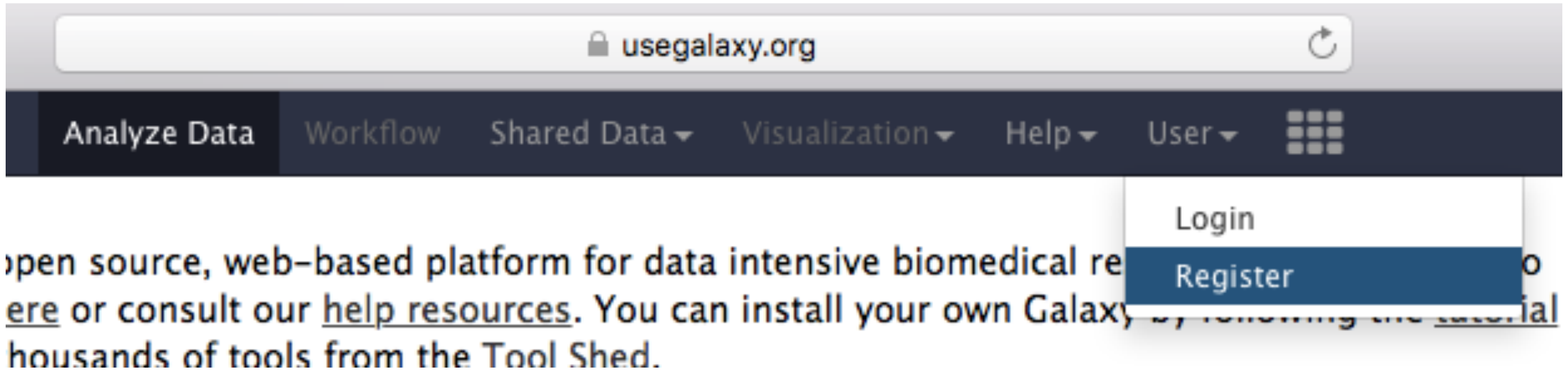

B. Open the link and import history

Click the link or copy it into your browser's location bar (e.g., bit.ly/ie-hiv). When page loads you will see the "import history" link. Click on it:

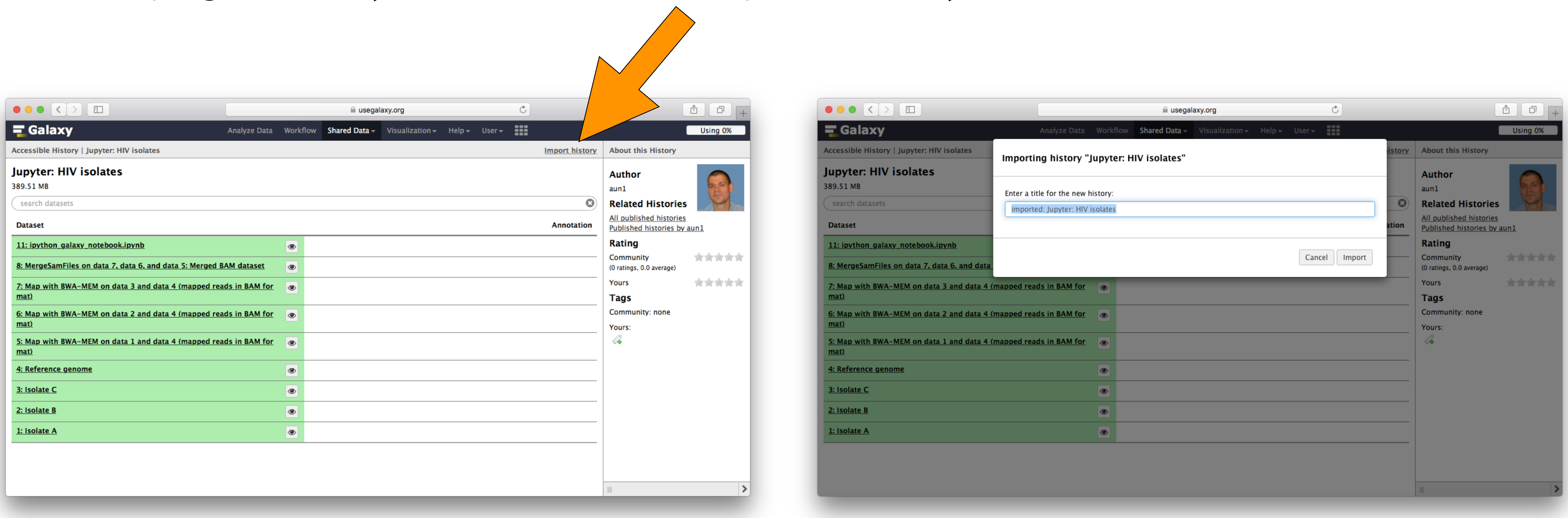

C. Start Jupyter

Once history is imported locate iPython/Jupyter dataset and expand it by clicking on it. Mouse over "Visualize" button and click "Jupyter" link:

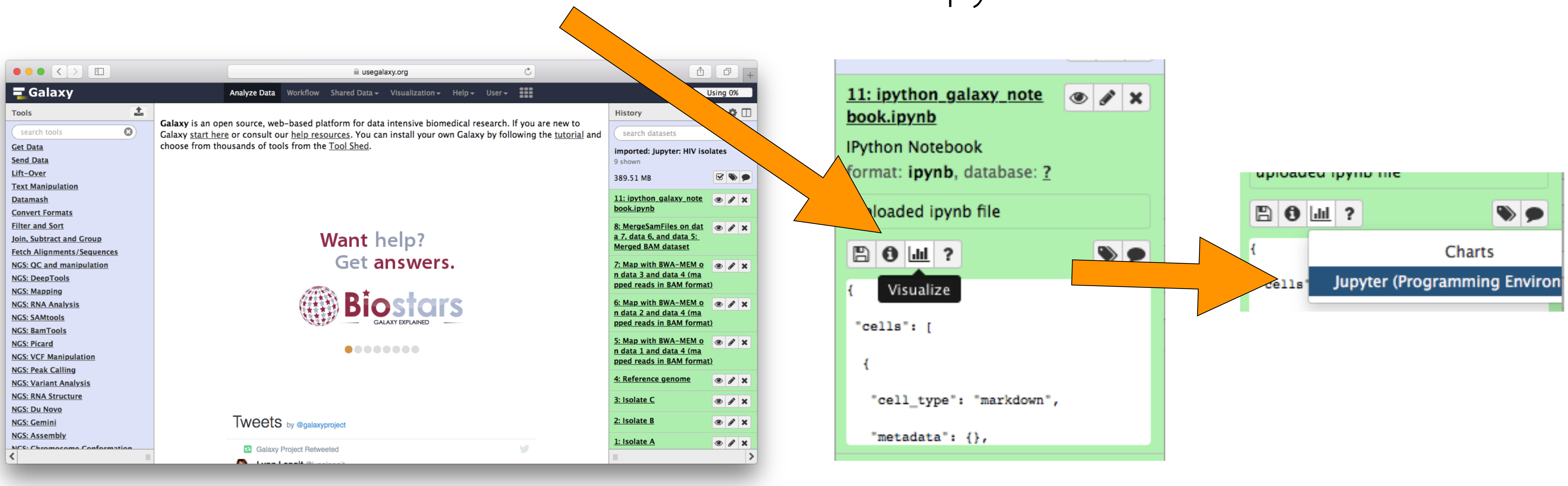

D. Instantiate Jupyter

Jupyter will start and you will be able to use the notebook. Questions? Use https://biostar.usegalaxy.org/ for get help.

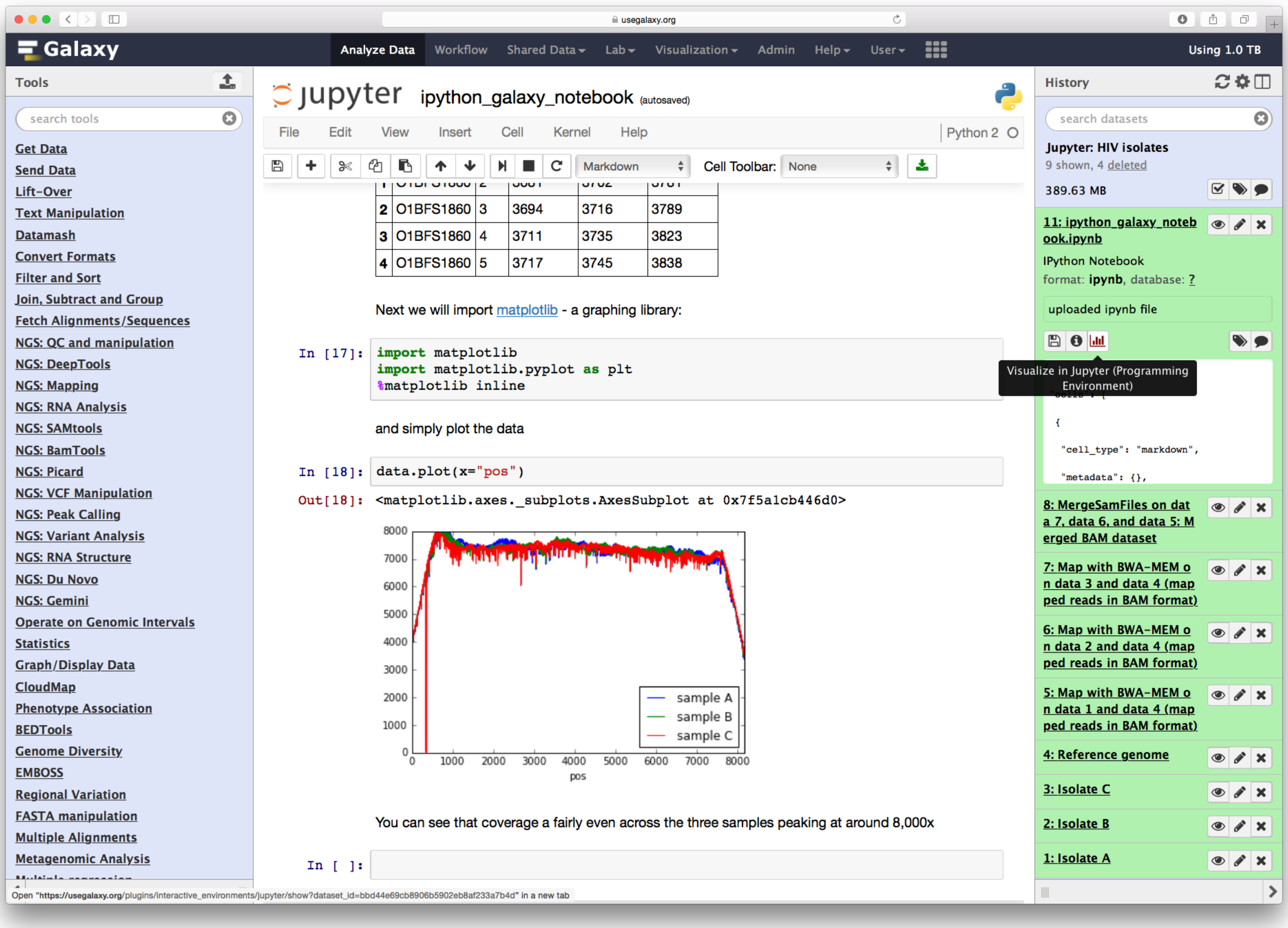

Supplement: S1 Fig — Go through steps highlighted in this figure to start Jupyter notebooks described in examples 1, 2, and 3. (PDF) [file pcbi.1005425.s001.pdf]
