# Supplementary material for: Jupyter and Galaxy: Easing entry barriers into complex data analyses for biomedical researchers
Source: PLoS Comput Biol. 2017 May 25;13(5):e1005425. doi: 10.1371/journal.pcbi.1005425 (PMC5444614; doi:10.1371/journal.pcbi.1005425)
Supplement: S1 File — (PDF) [file pcbi.1005425.s002.pdf]

ipython_galaxy_notebook

August 30, 2016

1 An interactive Galaxy Jupyter Notebook: Plotting read coverage distribution from a BAM file

You can access your data via the dataset number. For example, `handle = open(get(42), 'r')`. To save data, write your data to a file, and then call `put('filename.txt')`. The dataset will then be available in your galaxy history. Notebooks can be saved to Galaxy by clicking the large green button at the top right of the IPython interface. More help and informations can be found on the project website.

1.1 Getting data in

After starting Jupyter session we are copying a dataset from Galaxy history space into Jupyter space. In this particular case the dataset we need have number `8` in Galaxy's history. We set `bam_file` to `8`.

If you want to use a different dataset as input -> replace `8` with the number of that dataset

```
In [1]: bam_file = 8
```

```
In [2]: get(bam_file)
```

```
Out[2]: '/import/8'
```

1.2 Renaming dataset

`get` command names files using their galaxy history id. This is not a very meaningful name. So we will use UNIX commend `mv` to give this file a different name: `hiv_mapping.bam` (here `!` is used to tell Jupyter that we want to execute a shell command):

```
In [3]: !mv {bam_file} hiv_mapping.bam
```

1.3 Configuring bioconda

The easiest way to compute the depth of sequencing coverage is with `samtools`. However, they need to be installed. In turn, the easiest way to install `samtools` is with conda - a new generation package manager system. Here we are configuring conda's biological software channel bioconda

```
In [4]: !conda config --add channels bioconda
```

```
Warning: 'bioconda' already in 'channels' list, moving to the front
```

1.4 Installing `samtools`

And once conda is configures installing `samtools` is as easy as this (note the `-y` option required here):

```
In [5]: !conda install -y samtools
```

```
Fetching package metadata ...
Solving package specifications: ...

Package plan for installation in environment /opt/conda:

The following packages will be downloaded:

    package                    |            build
    ---------------------------|-----------------
    conda-env-2.5.2            |           py34_0          27 KB

The following packages will be UPDATED:

    conda-env: 2.5.1-py34_0 --> 2.5.2-py34_0

Fetching packages ...
conda-env-2.5. 100% |################################| Time: 0:00:00 274.92 kB/s
Extracting packages ...
[      COMPLETE      ]|##################################################| 100%
Unlinking packages ...
[      COMPLETE      ]|##################################################| 100%
Linking packages ...
[      COMPLETE      ]|##################################################| 100%
```

1.5 Split bam file on readgroups

In this example the bam file combines mapped reads derived from three different expriments. The relationship between each read and a corresponding sample is maintained with read group tags. Since we want to compute depth of coverage for each sample separately, we first need to split the original bam file into by read groups. This is done using `samtools split` command:

```
In [6]: !samtools split hiv_mapping.bam
```

Listing files will show three newly generated datasetes called hiv_mapping_0, *1, and* 2:

```
In [7]: !ls
```

```
9                  hiv_mapping_1.bam                readgroup_1.cvrg
all_cvrg.txt          hiv_mapping_2.bam                readgroup_2.cvrg
hiv_mapping.bam    ipython_galaxy_notebook.ipynb
hiv_mapping_0.bam  readgroup_0.cvrg
```

1.6 Computing coverage

We compute coverage using `samtools depth` command. Note how Python's `for` loop is used here. It iterates over three numbers (0, 1, and 2) generated by the `range` function. There are substituted into file names. This way we generate three coverage files (one for each read group) in one go.

Of particular importance is the use of `-a` option when we compute depth. Because of this flag `samtools depth` outputs depth at every position even is the coverage at that position is 0. This woild allow us to paste the three datasets side by side making sure that every row described the same position of the reference genome.

If your dataset has a different number of read groups -> replace `3` within the `range()` function with that number

```
In [8]: for item in range(3):
            !samtools depth -a hiv_mapping_{item}.bam > readgroup_{item}.cvrg
```

Listing files shows three additional datasets (ending with cvrg that we have just generated):

```
In [9]: !ls
```

```
9                     hiv_mapping_1.bam                  readgroup_1.cvrg
all_cvrg.txt            hiv_mapping_2.bam                  readgroup_2.cvrg
hiv_mapping.bam    ipython_galaxy_notebook.ipynb
hiv_mapping_0.bam  readgroup_0.cvrg
```

Now we can paste these three files side by side removing duplicate columns using UNIX `cut` command:

```
In [10]: !paste readgroup_* | cut -f 1,2,3,6,9 > all_cvrg.txt
```

This new datasets called `all_cvrg.txt` contains five columns (see outputs of `head` and `tail` commands below):

- Reference genome name
- Position in the genome
- Coverage in sample A
- Coverage in sample B
- Coverage in sample C

```
In [11]: !head -n 4 all_cvrg.txt
```

```
01BFS1860       1       3581        3577        3642
01BFS1860       2       3681        3702        3781
01BFS1860       3       3694        3716        3789
01BFS1860       4       3711        3735        3823
```

```
In [12]: !tail -n 4 all_cvrg.txt
```

```
01BFS1860       8159        3486        3524        3585
01BFS1860       8160        3454        3495        3543
01BFS1860       8161        3410        3456        3493
01BFS1860       8162        3383        3428        3476
```

1.7 Plotting coverage across the HIV genome

Let's import `pandas` a data manipulation library that allows datasets to be handled in an R-like way:

```
In [13]: import pandas as pd
```

We will then import the `all_cvrg.txt` dataset as Pandas dataframe:

```
In [14]: data = pd.read_table('all_cvrg.txt',header=None)
```

Set column names

```
In [15]: data.columns=["Ref","pos","sample A","sample B","sample C"]
```

And take a look at the result

```
In [16]: data.head()
```

Out[16]:

	Ref	pos	sample A	sample B	sample C
0	01BFS1860	1	3581	3577	3642
1	01BFS1860	2	3681	3702	3781
2	01BFS1860	3	3694	3716	3789
3	01BFS1860	4	3711	3735	3823
4	01BFS1860	5	3717	3745	3838

Next we will import matplotlib - a graphing library:

```
In [17]: import matplotlib
         import matplotlib.pyplot as plt
         %matplotlib inline
```

and simply plot the data

```
In [18]: data.plot(x="pos")
```

```
Out[18]: <matplotlib.axes._subplots.AxesSubplot at 0x7f5a1cb446d0>
```

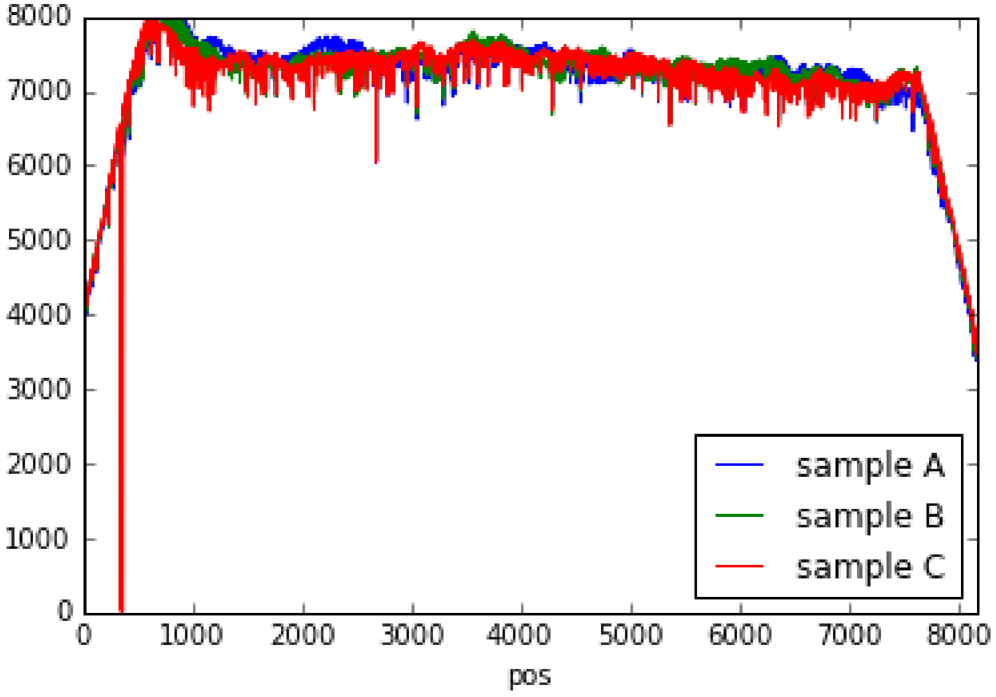

You can see that coverage a fairly even across the three samples peaking at around 8,000x

```
In [ ]:
```
