# Supplementary material for: Jupyter and Galaxy: Easing entry barriers into complex data analyses for biomedical researchers
Source: PLoS Comput Biol. 2017 May 25;13(5):e1005425. doi: 10.1371/journal.pcbi.1005425 (PMC5444614; doi:10.1371/journal.pcbi.1005425)
Supplement: S2 File — (PDF) [file pcbi.1005425.s003.pdf]

ipython_galaxy_notebook

August 30, 2016

1 Welcome to the interactive Galaxy IPython Notebook.

You can access your data via the dataset number. For example, `handle = open(get(42), 'r')`. To save data, write your data to a file, and then call `put('filename.txt')`. The dataset will then be available in your galaxy history. Notebooks can be saved to Galaxy by clicking the large green button at the top right of the IPython interface. More help and informations can be found on the project website.

1.1 Configure things

Before we begin we need to install a few things that we will be using. This Jupyter instance runs within your own private docker, so you can install anything. Let's start with loading Jupyter R extension `rpy2.ipython`:

```
In [1]: %load_ext rpy2.ipython
```

Next, we will install Bioconductor's DeSeq2 package using Conda, a powerful package manager:

```
In [2]: !conda install -y -c cgat bioconductor-deseq2=1.6.3
```

```
Fetching package metadata: ...
Solving package specifications: ...

Package plan for installation in environment /opt/conda:

The following packages will be downloaded:

    package                    |            build
    ---------------------------|-----------------
    sqlite-3.13.0              |                0         4.0 MB
    xz-5.2.2                   |                0         644 KB
    gsl-2.1                    |                1         7.3 MB
    libtiff-4.0.6              |                2         1.5 MB
    python-3.4.5               |                0        15.2 MB
    conda-env-2.5.1            |           py34_0          27 KB
    ruamel_yaml-0.11.14        |           py34_0         385 KB
    conda-4.1.11               |           py34_0         205 KB
    bioconductor-biocgenerics-0.12.1|                0         446 KB
    bioconductor-biobase-2.26.0|                0         2.0 MB
    bioconductor-s4vectors-0.4.0|                0         727 KB
    bioconductor-iranges-2.0.1 |                0         1.7 MB
    bioconductor-genomeinfodb-1.2.4|                0         413 KB
    bioconductor-xvector-0.6.0 |                0         389 KB
    bioconductor-genomic-ranges-1.18.4|                0         1.2 MB
    r-acepack-1.3_3.3          |        r3.2.2_1a          54 KB
    r-checkmate-1.5.1          |                0         254 KB
```

```
    r-formula-1.2_1           |        r3.2.2_0a         159 KB
    r-xml-3.98_1.3            |        r3.2.2_0a         1.6 MB
    r-bbmisc-1.9              |                0          268 KB
    r-gridextra-2.0.0         |        r3.2.2_0a         255 KB
    r-latticeextra-0.6_26     |        r3.2.2_2a         2.0 MB
    r-locfit-1.5_9.1          |                0         422 KB
    r-sendmailr-1.2_1         |                0          29 KB
    bioconductor-annotationdbi-1.28.1|                0         4.4 MB
    r-fail-1.2                |                0          41 KB
    bioconductor-annotate-1.44.0|                0         1.9 MB
    r-batchjobs-1.6           |           r3.2_0         407 KB
    bioconductor-biocparallel-1.0.3|                0         413 KB
    bioconductor-genefilter-1.48.1|                0         1.3 MB
    bioconductor-geneplotter-1.44.0|                0         1.4 MB
    r-hmisc-3.17_0            |        r3.2.2_0a         1.5 MB
    bioconductor-deseq2-1.6.3  |                0         1.3 MB
    ------------------------------------------------------------
                                           Total:        53.7 MB

The following NEW packages will be INSTALLED:

    bioconductor-annotate:       1.44.0-0
    bioconductor-annotationdbi:  1.28.1-0
    bioconductor-biobase:        2.26.0-0
    bioconductor-biocgenerics:   0.12.1-0
    bioconductor-biocparallel:   1.0.3-0
    bioconductor-deseq2:         1.6.3-0
    bioconductor-genefilter:     1.48.1-0
    bioconductor-geneplotter:    1.44.0-0
    bioconductor-genomeinfodb:   1.2.4-0
    bioconductor-genomic-ranges: 1.18.4-0
    bioconductor-iranges:        2.0.1-0
    bioconductor-s4vectors:      0.4.0-0
    bioconductor-xvector:        0.6.0-0
    gsl:                         2.1-1
    r-acepack:                   1.3_3.3-r3.2.2_1a
    r-batchjobs:                 1.6-r3.2_0
    r-bbmisc:                    1.9-0
    r-checkmate:                 1.5.1-0
    r-fail:                      1.2-0
    r-formula:                   1.2_1-r3.2.2_0a
    r-gridextra:                 2.0.0-r3.2.2_0a
    r-hmisc:                     3.17_0-r3.2.2_0a
    r-latticeextra:              0.6_26-r3.2.2_2a
    r-locfit:                    1.5_9.1-0
    r-sendmailr:                 1.2_1-0
    r-xml:                       3.98_1.3-r3.2.2_0a
    ruamel_yaml:                 0.11.14-py34_0

The following packages will be UPDATED:

    conda:                       4.0.5-py34_0 --> 4.1.11-py34_0
    conda-env:                   2.4.5-py34_0 --> 2.5.1-py34_0
    libtiff:                     4.0.6-1      --> 4.0.6-2
```

```
    python:                      3.4.4-0      --> 3.4.5-0
    sqlite:                      3.9.2-0      --> 3.13.0-0
    xz:                          5.0.5-1      --> 5.2.2-0

Fetching packages ...
sqlite-3.13.0- 100% |################################| Time: 0:00:00   5.06 MB/s
xz-5.2.2-0.tar 100% |################################| Time: 0:00:00   1.24 MB/s
gsl-2.1-1.tar. 100% |################################| Time: 0:00:00   8.26 MB/s
libtiff-4.0.6- 100% |################################| Time: 0:00:00   2.56 MB/s
python-3.4.5-0 100% |################################| Time: 0:00:01  11.59 MB/s
conda-env-2.5. 100% |################################| Time: 0:00:00 830.87 kB/s
ruamel_yaml-0. 100% |################################| Time: 0:00:00   1.08 MB/s
conda-4.1.11-p 100% |################################| Time: 0:00:00 795.80 kB/s
bioconductor-b 100% |################################| Time: 0:00:00   1.54 MB/s
bioconductor-b 100% |################################| Time: 0:00:00   2.50 MB/s
bioconductor-s 100% |################################| Time: 0:00:00   1.33 MB/s
bioconductor-i 100% |################################| Time: 0:00:00   2.51 MB/s
bioconductor-g 100% |################################| Time: 0:00:00   1.27 MB/s
bioconductor-x 100% |################################| Time: 0:00:00   1.19 MB/s
bioconductor-g 100% |################################| Time: 0:00:00   2.02 MB/s
r-acepack-1.3_ 100% |################################| Time: 0:00:00 831.66 kB/s
r-checkmate-1. 100% |################################| Time: 0:00:00 975.28 kB/s
r-formula-1.2_ 100% |################################| Time: 0:00:00 815.59 kB/s
r-xml-3.98_1.3 100% |################################| Time: 0:00:00   2.17 MB/s
r-bbmisc-1.9-0 100% |################################| Time: 0:00:00 930.82 kB/s
r-gridextra-2. 100% |################################| Time: 0:00:00 883.99 kB/s
r-latticeextra 100% |################################| Time: 0:00:00   3.17 MB/s
r-locfit-1.5_9 100% |################################| Time: 0:00:00   1.31 MB/s
r-sendmailr-1. 100% |################################| Time: 0:00:00 891.51 kB/s
bioconductor-a 100% |################################| Time: 0:00:00   5.50 MB/s
r-fail-1.2-0.t 100% |################################| Time: 0:00:00 423.72 kB/s
bioconductor-a 100% |################################| Time: 0:00:00   2.90 MB/s
r-batchjobs-1. 100% |################################| Time: 0:00:00   1.13 MB/s
bioconductor-b 100% |################################| Time: 0:00:00   1.42 MB/s
bioconductor-g 100% |################################| Time: 0:00:00   1.99 MB/s
bioconductor-g 100% |################################| Time: 0:00:00   2.48 MB/s
r-hmisc-3.17_0 100% |################################| Time: 0:00:00   2.44 MB/s
bioconductor-d 100% |################################| Time: 0:00:00   1.84 MB/s
Extracting packages ...
[      COMPLETE      ]|###################################################| 100%
Unlinking packages ...
[      COMPLETE      ]|###################################################| 100%
Linking packages ...
[      COMPLETE      ]|###################################################| 100%
```

Finally we will load two python libraries: `pandas` and `os`. `Pandas` will allow us to easily manipulate tabular datasets and `os` will be needed to parse file names.

```
In [3]: import pandas as pd
        import os
```

2 Import data from Galaxy history

To import datasets from Galaxy's history into Jupyter we will use dataset numbers. In this particular case we have four datasets: WT1, WT2, SNF2_1, and SNF2_2. We pass history numbers of these datasets into a

Python list as [10,12,14,16]. In your particular case these numbers **may be different** for change them accordingly. Take care to have wildtype (WT) datasets first and mutanats (SNF2) last:

```
In [4]: my_datasets = [10,12,14,16]
```

Now we iterate over this list and use get() function to copy each dataset into Jupyter

```
In [5]: for dataset in my_datasets:
            get(dataset)
```

Use shell ls command should show files names 10,12 . . . and so on (of course if you used different numbers they will be named differently):

```
In [6]: !ls

10  12        14  16        19  ipython_galaxy_notebook.ipynb
```

Below we define a function make_df that create a dataframe (the code is from notsoconsusing):

```
In [7]: def make_df(filename):
            df = pd.read_table(filename)
            name = (os.path.basename(filename)).split('.')[0]
            df.columns = [name, name+'_ct']
            return df
```

Now we use the above function to generate dataframes for each dataset in the list my_datasets:

```
In [8]: dfs = [make_df(str(dataset)) for dataset in my_datasets]
```

Now we are joining these datasets on gene names to have a table where each row is a gene and each column contains read counts for thois gene in each dataset (this function is from notsoconsusing):

```
In [9]: def join_dfs(ldf, rdf):
            return ldf.join(rdf, how='inner')

        final_df = reduce(join_dfs, dfs) #that's the magic
        final_df.head()
```

Out[9]:

	10	10_ct	12	12_ct	14	14_ct	16	16_ct
0	Q0017	0	Q0017	0	Q0017	0	Q0017	0
1	Q0032	0	Q0032	0	Q0032	0	Q0032	0
2	Q0045	0	Q0045	1	Q0045	0	Q0045	5
3	Q0050	2	Q0050	1	Q0050	5	Q0050	1
4	Q0055	0	Q0055	5	Q0055	3	Q0055	1

You will see that gene names are redundant and only the first can be saved. Below we create a list that would contain the numbers (ids) of columns to be removed:

```
In [10]: columns = []
         for i in range(len(list(final_df))):
             if ( i > 1 and i%2 == 0 ):
                 columns.append(i)
```

And the columns tobe removed are:

```
In [11]: columns

Out[11]: [2, 4, 6]
```

Now we will actually drop them and rename the first column as `gene_id`:

```
In [12]: counts = final_df.drop(final_df.columns[columns], axis=1)
         counts = counts.rename(columns={ list(counts)[0]: "gene_id"})
         counts.head()
```

```
Out[12]:   gene_id  10_ct  12_ct  14_ct  16_ct
         0   Q0017      0      0      0      0
         1   Q0032      0      0      0      0
         2   Q0045      0      1      0      5
         3   Q0050      2      1      5      1
         4   Q0055      0      5      3      1
```

The rest of the data analysis will be performed in `R`, so we will input Pandas dataframe `counts` into `R`:

```
In [13]: %R -i counts
```

2.1 Massaging the data

Let's take a look at the first several row of this dataframe:

```
In [14]: %%R
         head(counts)
```

```
  gene_id X10_ct X12_ct X14_ct X16_ct
0   Q0017      0      0      0      0
1   Q0032      0      0      0      0
2   Q0045      0      1      0      5
3   Q0050      2      1      5      1
4   Q0055      0      5      3      1
5   Q0060      0      0      0      0
```

Next, we will use this R expression to name all rows after gene names. In this case the gene names are stored in column "`gene_id`" hence the `<- counts$gene_id` expression:

```
In [15]: %%R
         row.names(counts) <- counts$gene_id
```

And now if we look at the few rows of `read.counts` dataframe we will see that rows are named not 1, 2, 3, etc but Q0010, Q0017 etc:

```
In [16]: %%R
         head(counts)
```

```
      gene_id X10_ct X12_ct X14_ct X16_ct
Q0017   Q0017      0      0      0      0
Q0032   Q0032      0      0      0      0
Q0045   Q0045      0      1      0      5
Q0050   Q0050      2      1      5      1
Q0055   Q0055      0      5      3      1
Q0060   Q0060      0      0      0      0
```

Now we will remove the column containing names using not so intuitive R syntax below:

```
In [17]: %%R
         counts <- counts[ , -c(1)]
```

Let's check if it is actually removed:

```
In [18]: %%R
         head(counts)
```

```
      X10_ct X12_ct X14_ct X16_ct
Q0017      0      0      0      0
Q0032      0      0      0      0
Q0045      0      1      0      5
Q0050      2      1      5      1
Q0055      0      5      3      1
Q0060      0      0      0      0
```

Give sensible names (conditions) to columns within the dataframe:

```
In [19]: %%R
         names(counts) <- c("WT_1", "WT_2", "SNF2_1", "SNF2_2")
```

Check that this is in fact the case:

```
In [20]: %%R
         head(counts)
```

```
      WT_1 WT_2 SNF2_1 SNF2_2
Q0017    0    0      0      0
Q0032    0    0      0      0
Q0045    0    1      0      5
Q0050    2    1      5      1
Q0055    0    5      3      1
Q0060    0    0      0      0
```

Here we will create a new dataframe called `read.count_noZero` by removing all rows from the original dataframe (`read.counts`) where read counts are zero for all conditions:

```
In [21]: %%R
         rc.keep <- rowSums(counts) > 0
         read.counts_noZero <- counts[rc.keep,]
```

No rows with all zeros are now visible in the first few lines of the data:

```
In [22]: %%R
         head(read.counts_noZero)
```

```
      WT_1 WT_2 SNF2_1 SNF2_2
Q0045    0    1      0      5
Q0050    2    1      5      1
Q0055    0    5      3      1
Q0085    0    1      1      0
Q0105    0    1      0      0
Q0120    0    0      2      0
```

2.2 Normalizing counts with DESeq2

Tell R to use DESeq2 library:

```
In [23]: %%R
         library ( DESeq2 )
```

```
/opt/conda/envs/python2/lib/python2.7/site-packages/rpy2/robjects/functions.py:106: UserWarning: Loadin

  res = super(Function, self).__call__(*new_args, **new_kwargs)
/opt/conda/envs/python2/lib/python2.7/site-packages/rpy2/robjects/functions.py:106: UserWarning: Loadin

  res = super(Function, self).__call__(*new_args, **new_kwargs)
/opt/conda/envs/python2/lib/python2.7/site-packages/rpy2/robjects/functions.py:106: UserWarning: Loadin

  res = super(Function, self).__call__(*new_args, **new_kwargs)
/opt/conda/envs/python2/lib/python2.7/site-packages/rpy2/robjects/functions.py:106: UserWarning: Loadin

  res = super(Function, self).__call__(*new_args, **new_kwargs)
/opt/conda/envs/python2/lib/python2.7/site-packages/rpy2/robjects/functions.py:106: UserWarning:
Attaching package: 'BiocGenerics'


  res = super(Function, self).__call__(*new_args, **new_kwargs)
/opt/conda/envs/python2/lib/python2.7/site-packages/rpy2/robjects/functions.py:106: UserWarning: The fo

    clusterApply, clusterApplyLB, clusterCall, clusterEvalQ,
    clusterExport, clusterMap, parApply, parCapply, parLapply,
    parLapplyLB, parRapply, parSapply, parSapplyLB


  res = super(Function, self).__call__(*new_args, **new_kwargs)
/opt/conda/envs/python2/lib/python2.7/site-packages/rpy2/robjects/functions.py:106: UserWarning: The fo

    xtabs


  res = super(Function, self).__call__(*new_args, **new_kwargs)
/opt/conda/envs/python2/lib/python2.7/site-packages/rpy2/robjects/functions.py:106: UserWarning: The fo

    Filter, Find, Map, Position, Reduce, anyDuplicated, append,
    as.data.frame, as.vector, cbind, colnames, do.call, duplicated,
    eval, evalq, get, intersect, is.unsorted, lapply, mapply, match,
    mget, order, paste, pmax, pmax.int, pmin, pmin.int, rank, rbind,
    rep.int, rownames, sapply, setdiff, sort, table, tapply, union,
    unique, unlist, unsplit


  res = super(Function, self).__call__(*new_args, **new_kwargs)
/opt/conda/envs/python2/lib/python2.7/site-packages/rpy2/robjects/functions.py:106: UserWarning: Loadin

  res = super(Function, self).__call__(*new_args, **new_kwargs)
/opt/conda/envs/python2/lib/python2.7/site-packages/rpy2/robjects/functions.py:106: UserWarning: Loadin

  res = super(Function, self).__call__(*new_args, **new_kwargs)
```

```
/opt/conda/envs/python2/lib/python2.7/site-packages/rpy2/robjects/functions.py:106: UserWarning: Loading

  res = super(Function, self).__call__(*new_args, **new_kwargs)
/opt/conda/envs/python2/lib/python2.7/site-packages/rpy2/robjects/functions.py:106: UserWarning: Loading

  res = super(Function, self).__call__(*new_args, **new_kwargs)
/opt/conda/envs/python2/lib/python2.7/site-packages/rpy2/robjects/functions.py:106: UserWarning: Loading

  res = super(Function, self).__call__(*new_args, **new_kwargs)
```

Now for DESeq2 to work we need to create another dataframe that maps sample name to a conditions. In our example here we have two conditions: * WT = wildtype * SNF2 = snf2

Each of these conditions has two samples (replicates): WT1 and WT2 as well as SNF2_1 and SNF2_2. So we will create a new dataframe called `sample.info` that will hold this information:

```
In [24]: %%R
         sample.info <- data.frame(condition = c( rep("WT", 2), rep("SNF2", 2)),
                                   row.names = c(paste("WT", c(1:2), sep="_"),
                                                 paste("SNF2", c(1:2),sep="_")) )
```

```
In [25]: %%R
         head(sample.info)
```

```
       condition
WT_1          WT
WT_2          WT
SNF2_1      SNF2
SNF2_2      SNF2
```

We need to generate DESeq2 dataset:

```
In [26]: %%R

         DESeq.ds <- DESeqDataSetFromMatrix( countData = read.counts_noZero ,
                                             colData = sample.info , design = ~ condition )
```

The following command will sum read counts for all four samples and thus inform us about the library sizes:

```
In [27]: %R colSums ( read.counts_noZero )

Out[27]: array([ 5034929.,  6725982.,  8884141.,  7502269.])
```

Here we will let DESeq2 to performs its statistical magic by estimating the size factors that will be used in normalization:

```
In [28]: %R DESeq.ds <- estimateSizeFactors ( DESeq.ds )

Out[28]: <RS4 - Python:0x7f1e6b8472d8 / R:0xa6a85d0>
```

Now we will perform the normalization:

```
In [29]: %R counts.normalized <- counts ( DESeq.ds , normalized = TRUE )
```

```
Out[29]: array([[  0.        ,   1.0284004 ,   0.        ,   4.37455011],
                [  3.23646129,   1.0284004 ,   3.40192088,   0.87491002],
                [  0.        ,   5.14200198,   2.04115253,   0.87491002],
                ...,
                [ 71.20214841,  75.07322897,  69.39918598,  64.7433416 ],
                [  4.85469194,   4.11360159,   1.36076835,   1.74982004],
                [ 55.01984195,  25.71000992,  24.49383035,  30.62185076]])
```

And also perform log2 transformation:

```
In [30]: %R log.norm.counts <- log2( counts.normalized + 1)
```

```
Out[30]: array([[ 0.        ,  1.02034246,  0.        ,  2.42614399],
                [ 2.08285969,  1.02034246,  2.13813322,  0.90682136],
                [ 0.        ,  2.61870898,  1.60461818,  0.90682136],
                ...,
                [ 6.17396986,  6.24931694,  6.13748684,  6.03877288],
                [ 2.54959326,  2.35433976,  1.23925649,  1.45933721],
                [ 5.80786601,  4.73930861,  4.67207624,  4.9828499 ]])
```

To appreciate the effect of log2 transformation let's plot non-transformed counts:

```
In [31]: %%R
         boxplot( counts.normalized , notch = TRUE ,
                 main = "untransformed read counts" , ylab = "read counts" )
```

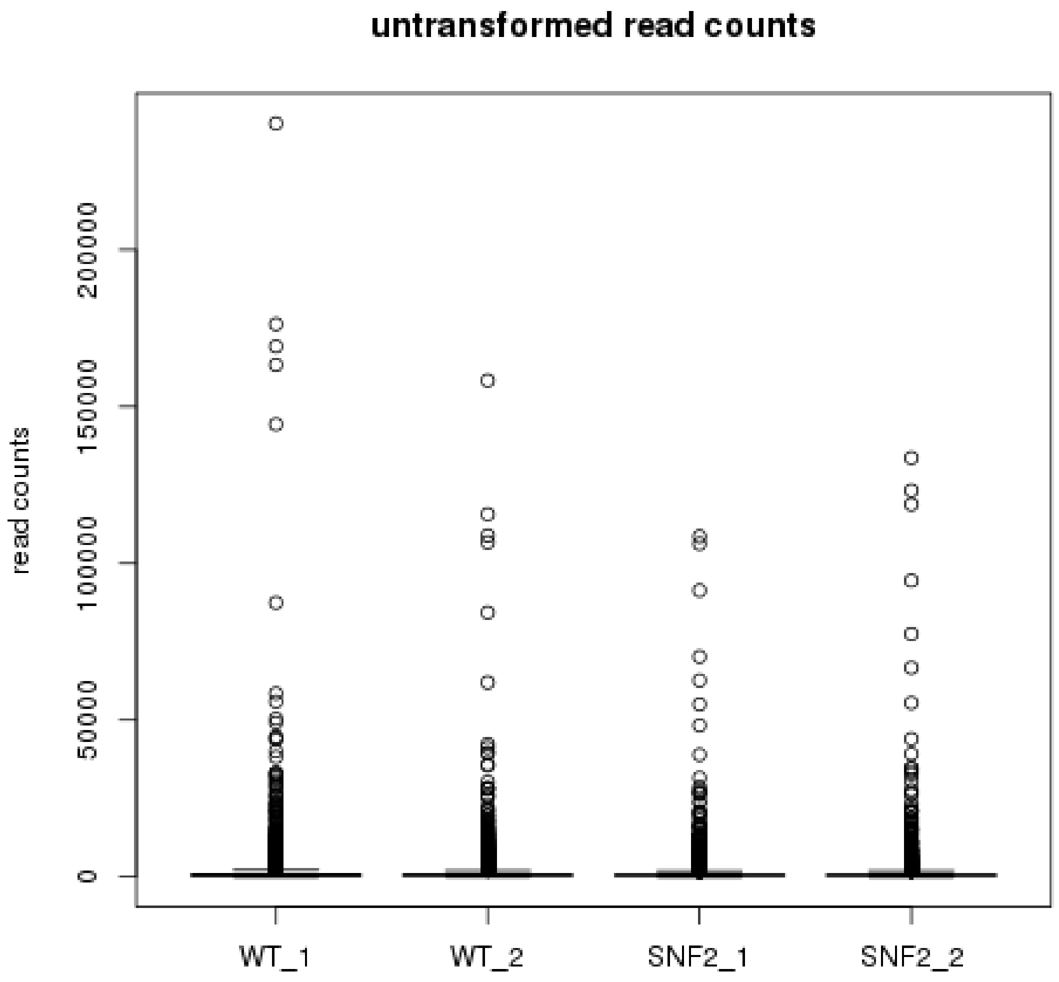

followed by transformed ones:

```
In [32]: %%R
         boxplot( log.norm.counts , notch = TRUE ,
                 main = "log2-transformed read counts" , ylab = "log2(read counts)" )
```

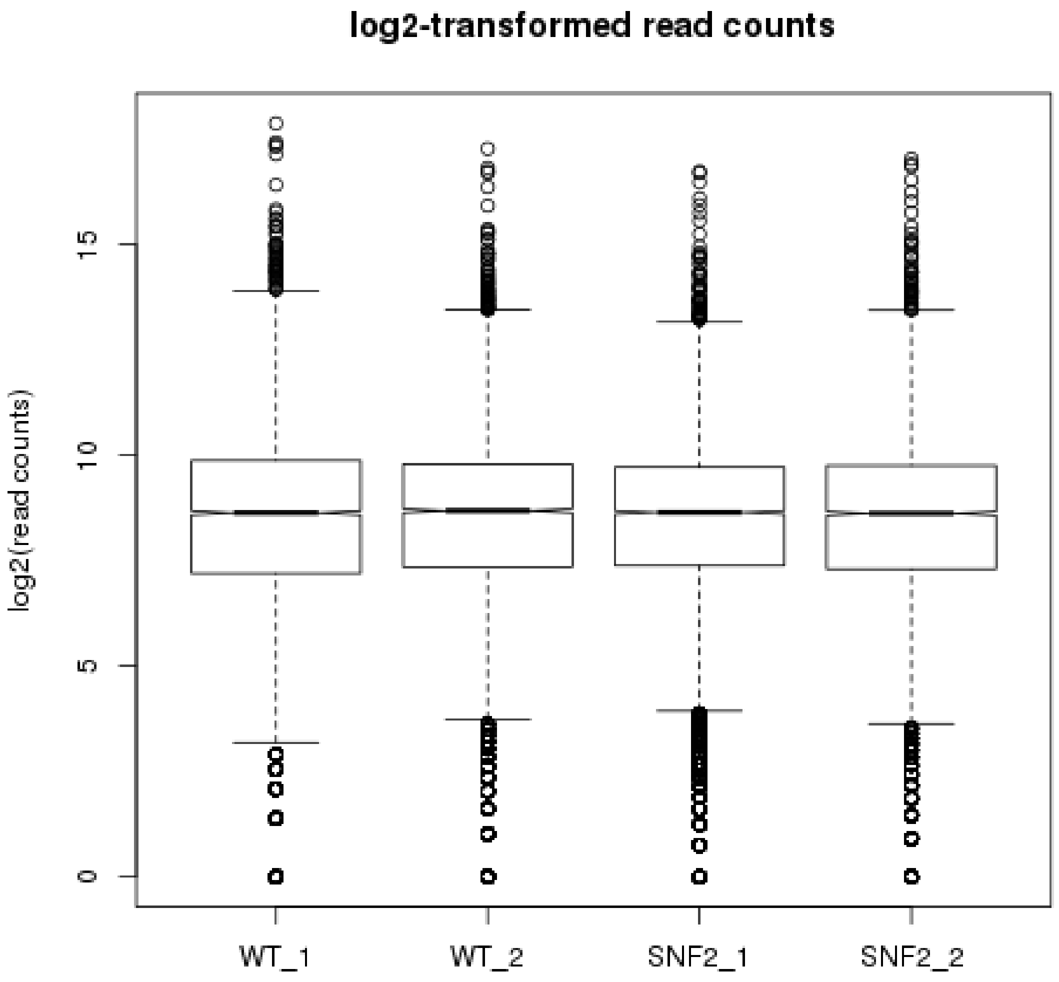

2.3 Shrinking varinance

Read count data is heteroscedastic. In this particular case there a greater variance among expression estimates for genes where read count is low. This can be visualized by plotting log2 normalized contans for two conditions against each other:

```
In [33]: %%R
         plot (log.norm.counts [ ,1:2] , cex =.1 , main = "Normalized log2 ( read counts )" )
```

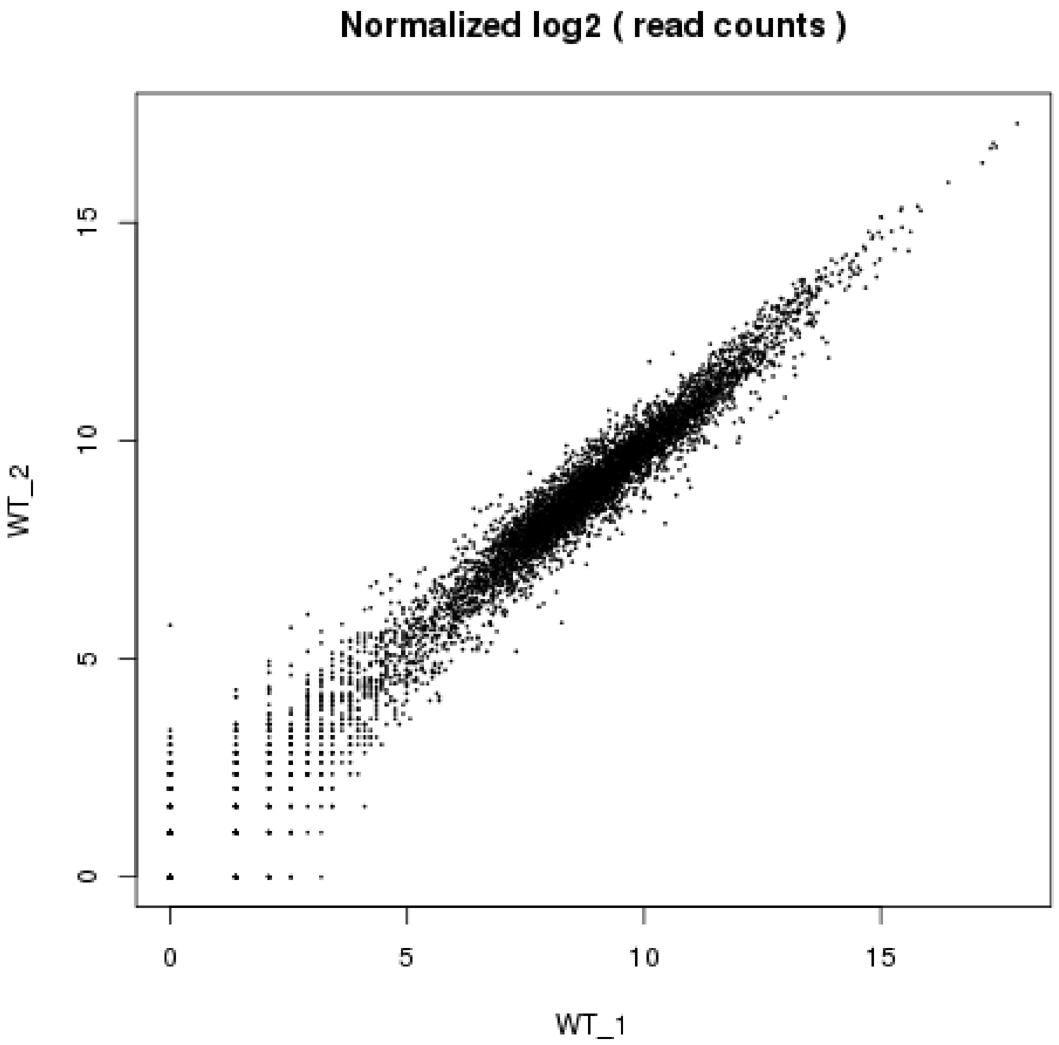

The DESeq2 allows to adjust for heteroscedasticity by assigning more homogeneous read to genes with low counts, so they resemble genes with high counts. This is performed using the `rlog` function:

In [34]:
```
%%R
rlog.DESeq.sumExp <- rlog( DESeq.ds , blind = TRUE )
rlog.norm.counts <- assay( rlog.DESeq.sumExp )
```

Now if we visualize the same relationship using `rlog` transformed counts we will see a much “narrower” plot:

In [35]:
```
%%R
plot (rlog.norm.counts [ ,1:2] , cex =.1 , main = "Normalized rlog transformed count ( read co
```

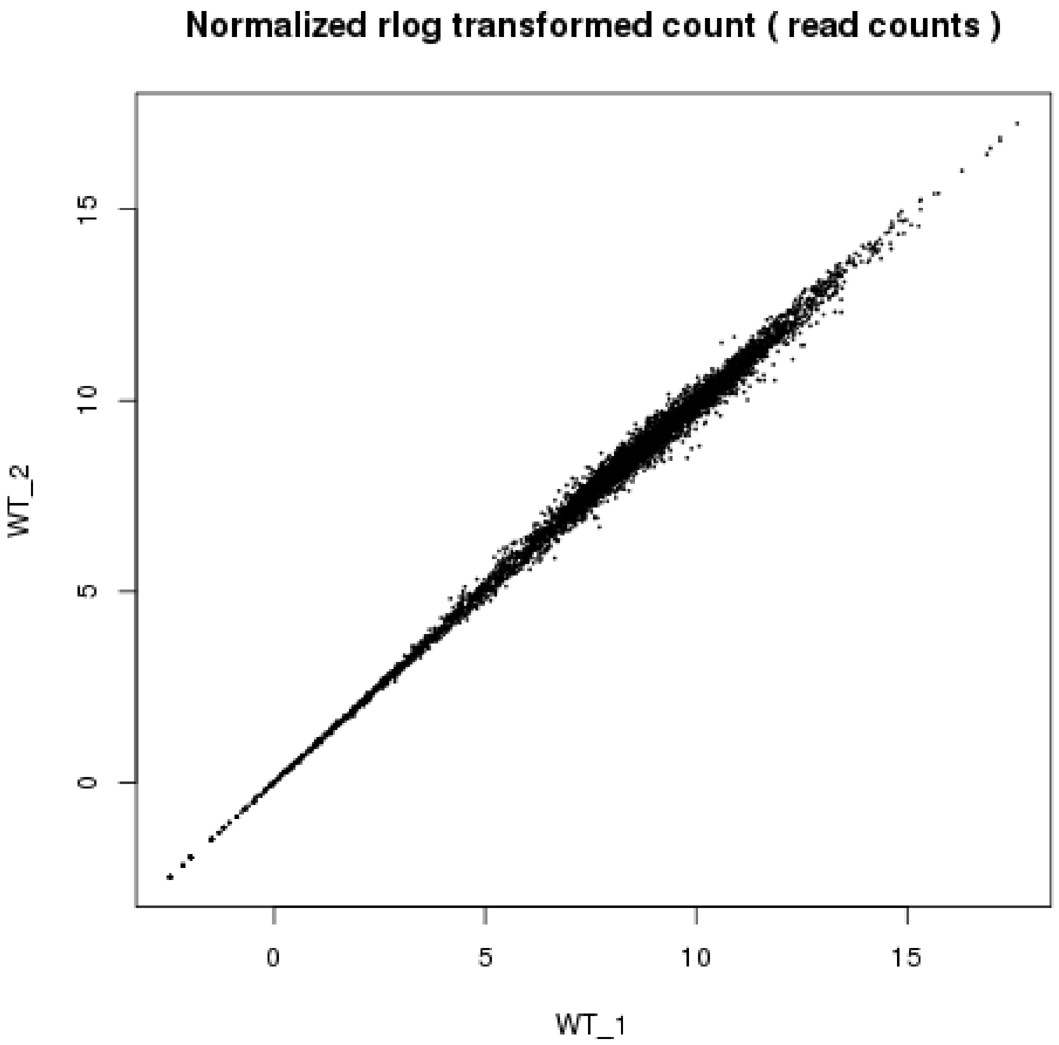

And so get the idea. You can continue exploring these (or other) data using this Jupyter notebook as a staring point.

In [ ]:
