# Supplementary material for: Jupyter and Galaxy: Easing entry barriers into complex data analyses for biomedical researchers
Source: PLoS Comput Biol. 2017 May 25;13(5):e1005425. doi: 10.1371/journal.pcbi.1005425 (PMC5444614; doi:10.1371/journal.pcbi.1005425)
Supplement: S3 File — (PDF) [file pcbi.1005425.s004.pdf]

ipython_galaxy_notebook

August 30, 2016

1 Maternal Age Effect and Severe Germline bottleneck in the Inheritance of mitochondrial DNA heteroplasmy

This notebook replicates the analyses shown in Rebolledo-Jaramillo, Su et al (2014) *Maternal Age Effect and Severe Germline bottleneck in the Inheritance of mitochondrial DNA heteroplasmy* **PNAS October 28, 2014 vol. 111 no. 43 15474-15479**

This analysis uses the following datasets as inputs:

- Allele counts produced with Galaxy pipeline (TODO: Provide link to Galaxy history here);
- GenBank file containing sequence and annotation for human mitochondrial genome (accession `NC_012920.1`);
- Tab-delimited file of ages for individuals analyzed here
- Known list of problematic sites to be excluded from the analysis

2 Define inputs

This notebook requires three input datasets: * List of variable sites * List of ages for all individuals * List of bad sites (see "Define problematic sites and regions" below)

```
In [1]: # Replace '1413' with the number of Galaxy history item
        # containing **Varinable sites**
        var_sites = 1413

        # Replace '1414' with the number of Galaxy history item
        # containing **Sample ages**
        ages = 1414

        # Replace '1415' with the number of Galaxy history item
        # containing **Bad sites**
        bad_sites = 1415
```

2.1 Import necessary python modules

- pandas - A library providing high-performance, easy-to-use data structures and data analysis tools
- numpy - A package for scientific computing with Python
- itertools - Functions for creation of iterators for efficient looping
- biopython - A set of Python modules for biological computation

```
In [2]: import pandas as pd
        import numpy as np
        import itertools
        from Bio import SeqIO
```

```
from Bio.Seq import Seq
from Bio import Entrez
from Bio.Alphabet import IUPAC
```

2.2 Load R extensions and install necessary R modules

```
In [3]: # Load R magic, which will allow running R directly in the notebook
        %load_ext rpy2.ipython
```

```
In [4]: # Make a directory where R modules will be installed
        !mkdir R
```

```
mkdir: cannot create directory 'R': File exists
```

```
In [5]: %%R
        install.packages("shape", lib="R", repos="http://cran.cnr.berkeley.edu")
```

```
/opt/conda/envs/python2/lib/python2.7/site-packages/rpy2/robjects/functions.py:106: UserWarning: trying

  res = super(Function, self).__call__(*new_args, **new_kwargs)
/opt/conda/envs/python2/lib/python2.7/site-packages/rpy2/robjects/functions.py:106: UserWarning: Conten
  res = super(Function, self).__call__(*new_args, **new_kwargs)
/opt/conda/envs/python2/lib/python2.7/site-packages/rpy2/robjects/functions.py:106: UserWarning:  lengt

  res = super(Function, self).__call__(*new_args, **new_kwargs)
/opt/conda/envs/python2/lib/python2.7/site-packages/rpy2/robjects/functions.py:106: UserWarning: =
  res = super(Function, self).__call__(*new_args, **new_kwargs)
/opt/conda/envs/python2/lib/python2.7/site-packages/rpy2/robjects/functions.py:106: UserWarning:

  res = super(Function, self).__call__(*new_args, **new_kwargs)
/opt/conda/envs/python2/lib/python2.7/site-packages/rpy2/robjects/functions.py:106: UserWarning: downlo


  res = super(Function, self).__call__(*new_args, **new_kwargs)


The downloaded source packages are in
'/tmp/RtmpZwnpqq/downloaded_packages'
```

```
In [6]: %%R
        install.packages("sm", lib="R", repos="http://cran.cnr.berkeley.edu")
```

```
/opt/conda/envs/python2/lib/python2.7/site-packages/rpy2/robjects/functions.py:106: UserWarning: trying

  res = super(Function, self).__call__(*new_args, **new_kwargs)
/opt/conda/envs/python2/lib/python2.7/site-packages/rpy2/robjects/functions.py:106: UserWarning:  lengt

  res = super(Function, self).__call__(*new_args, **new_kwargs)
/opt/conda/envs/python2/lib/python2.7/site-packages/rpy2/robjects/functions.py:106: UserWarning: downlo


  res = super(Function, self).__call__(*new_args, **new_kwargs)
```

```
The downloaded source packages are in
'/tmp/RtmpZwnpqq/downloaded_packages'
```

In [7]:
```
%%R
install.packages("vioplot", lib="R", repos="http://cran.cnr.berkeley.edu")
```

```
/opt/conda/envs/python2/lib/python2.7/site-packages/rpy2/robjects/functions.py:106: UserWarning: trying

  res = super(Function, self).__call__(*new_args, **new_kwargs)
/opt/conda/envs/python2/lib/python2.7/site-packages/rpy2/robjects/functions.py:106: UserWarning:  lengt

  res = super(Function, self).__call__(*new_args, **new_kwargs)
/opt/conda/envs/python2/lib/python2.7/site-packages/rpy2/robjects/functions.py:106: UserWarning: downlo


  res = super(Function, self).__call__(*new_args, **new_kwargs)


The downloaded source packages are in
'/tmp/RtmpZwnpqq/downloaded_packages'
```

In [8]:
```
%%R
require(shape, lib.loc="R")
require(sm, lib.loc="R")
require(vioplot, lib.loc="R")
```

```
/opt/conda/envs/python2/lib/python2.7/site-packages/rpy2/robjects/functions.py:106: UserWarning: Loadin

  res = super(Function, self).__call__(*new_args, **new_kwargs)
/opt/conda/envs/python2/lib/python2.7/site-packages/rpy2/robjects/functions.py:106: UserWarning: Loadin

  res = super(Function, self).__call__(*new_args, **new_kwargs)
/opt/conda/envs/python2/lib/python2.7/site-packages/rpy2/robjects/functions.py:106: UserWarning: Packag

  res = super(Function, self).__call__(*new_args, **new_kwargs)
/opt/conda/envs/python2/lib/python2.7/site-packages/rpy2/robjects/functions.py:106: UserWarning: Loadin

  res = super(Function, self).__call__(*new_args, **new_kwargs)
```

2.3 Load datasets

In this example all necessary data are located in Galaxy's history. They can be accessed using the `get()` function. For example, to load data in the first history item into Jupyter environment simply use `get(1)`, where 1 is the history item number.

Obviously, if your history looks different, change the numbers in the cells below.

In [9]:
```
# Load Allele Counts

with open(get(var_sites)) as ac:
    first_line = ac.readline()
    if first_line.startswith("#"):
```

```
        df = pd.read_table(ac)
    else:
        df = pd.read_table(ac,header=None)

# The line below prints the first twio lines of the data to give you an idea
df.head(2)
```

Out[9]:

```
         0     1  2     3  4  5     6     7  8  9    10     11  12 13 14  \
0  M117-bl  chrM  2  4343  0  0     0  5955  0  0     0  10298   1  A  .
1  M117-bl  chrM  3     0  0  0  4385     0  0  0  5888  10273   1  T  .

    15 16
0  0.0  .
1  0.0  .
```

In [10]:

```
# Load Genbank file containing mitochondrial genome sequence and annotations
# See http://biopython.org/DIST/docs/tutorial/Tutorial.html#htoc55
# This loads record with Accession:NC_012920.1  GI:251831106
# This cell should return "NC_012920.1 with 105 features"

# Code below loads directly from NCBI but requires Internet connection
# Alternatively you can load this dataset from history using the line below:
# rCRS = SeqIO.read(get(<HISTORY ITEM NUMBER>), "genbank")

Entrez.email = "A.N.Other@example.com"
handle = Entrez.efetch(db="nucleotide", rettype="gb", retmode="text", id="251831106")
rCRS = SeqIO.read(handle, "genbank")
handle.close()
print("%s with %i features" % (rCRS.id, len(rCRS.features)))
```

```
NC_012920.1 with 105 features
```

In [11]:

```
# Load individuals' ages (ages in days)

with open(get(ages)) as sa:
    first_line = sa.readline()
    if first_line.startswith("#"):
        sampAges = pd.read_table(sa)
    else:
        sampAges = pd.read_table(sa,header=None)
sampAges.head(2)
```

Out[11]:

	0	1	2	3
0	M132	16658	M132C1	7460
1	M137	14294	M137C2	6202

2.4 Define problematic sites and regions

- Problematic sites are defined as heteroplasmic sites that failed to be validated by experimental means. In particular, there is an additional screenning step not shown here, where we calculate the cycle bias of the site, i.e. whether the alternative allele is supported primarily by nucleotides within 25 bp of the read ends. There are 9 such cases, and two additional cases of sites we could not replicate with a new long range PCR (deemed PCR errors). These 11 sites are provided as an input dataset for this analysis.
- Problematic reagions include:

- mtDNA homopolymeres
- region around the artificial “N” at position 3107
- regions within 50 bp of the long range PCR primers

```
In [12]: # Read in bad (problematic) sites dataset from history

         knownBadhqSites = pd.read_table(get(bad_sites),header=None)
```

```
In [13]: # Define problematic regions

         mask = [(66,71),(303,311),(514,523),(12418,12425),(16184,16193),
               (3105,3109),(2817,2868),(3320,3370),(10796,10846),(11520,11570)]

         maskRegions = list()
         for start,end in mask:
             maskRegions+=range(start,end+1)
```

2.5 Prepare data

If a header was present in the allele counts input dataset, Pandas assigned the column names automatically. However we will standardize the column names so they can be easily accessed later.

```
In [14]: df.columns=["sample","reference","position","A","C","G","T","a","c","g","t","cvrg","nalleles",
```

```
In [15]: # Let's take a look at the first two lines in the data frame
         df.head(2)
```

```
Out[15]:     sample reference  position     A  C  G     T     a  c  g     t   cvrg  \
         0  M117-bl      chrM         2  4343  0  0     0  5955  0  0     0  10298
         1  M117-bl      chrM         3     0  0  0  4385     0  0  0  5888  10273

            nalleles major minor  maf  sb
         0         1     A     .  0.0  .
         1         1     T     .  0.0  .
```

In our data, all but one mother-child pair conforms to the naming convention:

mother	child
family-tissue	familyChild#-tissue
M477-ch	M477C1-ch

However, the pair M502G (grandmother) and M501 (mother) break the rule. So, we adjusted their ids accordingly:

```
In [16]: old = ["M502G-ch","M502G-bl","M501-ch","M501-bl"]
         new = ["M502-ch","M502-bl","M502C1-ch","M502C1-bl"]
         df.replace(to_replace=old,value=new,inplace=True)
```

2.6 Plot sequencing depth distribution (Fig. S7)

At this point we can calculate the coverage distribution of each sample, as shown in Figure S7 in the PNAS paper. To do so, we need to split the dataframe into blood and cheek dataframes, and make the object available to R (via Rpy2).

```
In [17]: # Here we split the dataframe into blood and cheek samples
         blood = df[df['sample'].str.contains("-bl")]
         cheek = df[df['sample'].str.contains("-ch")]
```

```
In [18]: # Let's look at blood data frame
         blood.head(2)
```

```
Out[18]:     sample reference  position     A  C  G     T     a  c  g     t   cvrg  \
         0  M117-bl      chrM         2  4343  0  0     0  5955  0  0     0  10298
         1  M117-bl      chrM         3     0  0  0  4385     0  0  0  5888  10273

            nalleles major minor  maf sb
         0         1     A     .  0.0  .
         1         1     T     .  0.0  .
```

```
In [19]: # And at the cheek data frame
         cheek.head(2)
```

```
Out[19]:         sample reference  position     A  C     G  T     a  c     g  t  cvrg  \
         16560  M117-ch      chrM         1     0  0  1829  0     0  0  3239  0  5068
         16561  M117-ch      chrM         2  1829  0     0  0  3291  0     0  0  5120

                nalleles major minor  maf sb
         16560         1     G     .  0.0  .
         16561         1     A     .  0.0  .
```

```
In [20]: # Use Rmagic to load data into R using the -i flag
         # This step will take a bit (~2 min)

         %R -i cheek,blood
```

Let's peek at the R version of the blood dataframe:

```
In [21]: %%R
         head(blood,2)

   sample reference position    A C G    T    a c g    t  cvrg nalleles major
0 M117-bl      chrM        2 4343 0 0    0 5955 0 0    0 10298        1     A
1 M117-bl      chrM        3    0 0 0 4385    0 0 0 5888 10273        1     T
  minor maf sb
0     .   0  .
1     .   0  .
```

Transform numeric looking columns into actual numeric columns to guarantee the value types:

```
In [22]: %%R
         tonumeric = c(3:13,16)
         blood[,tonumeric] = apply(blood[,tonumeric], 2, function(x) as.numeric(as.character(x)))
         cheek[,tonumeric] = apply(cheek[,tonumeric], 2, function(x) as.numeric(as.character(x)))
```

Define custom R function to generate **Figure S7**:

```
In [23]: %%R
         boxPlotCvrg = function(data,tissue){
```

```
names = sort(unique(data[["sample"]]))
data[["sample"]] = factor(data[["sample"]],levels=names)

boxplot(log10(cvrg)~sample,data=data,whisklty="solid",outline=F,
        whisklwd=0.5,boxlwd=1,medlwd=1,medcol="red",main="",
        ylab="log10(coverage)",bty="n",frame=F,boxcol="white",
        boxfill="black",medlwd=3,whiskcol="grey",staplecol="grey",ylim=c(2,6))

mtext(tissue,adj=0,side=3,las=1,at=length(names)/2,font=2,cex=1.25)
}
```

2.6.1 Plot the figure

You can adjust the size of the plotting image by adjusting:

- `-w` = width
- `-h` = height
- `-u` = units
- `-r` = resolution

In [24]:
```
%%R -w 18 -h 10 -u in -r 72

par(las=2)
par(mar=c(4,4,4,1))
par(oma=c(2,2,0,0))
par(mfrow=c(2,1))
par(cex.lab=1.25)
par(cex.axis=0.75)

boxPlotCvrg(cheek,"cheek")
boxPlotCvrg(blood,"blood")
```

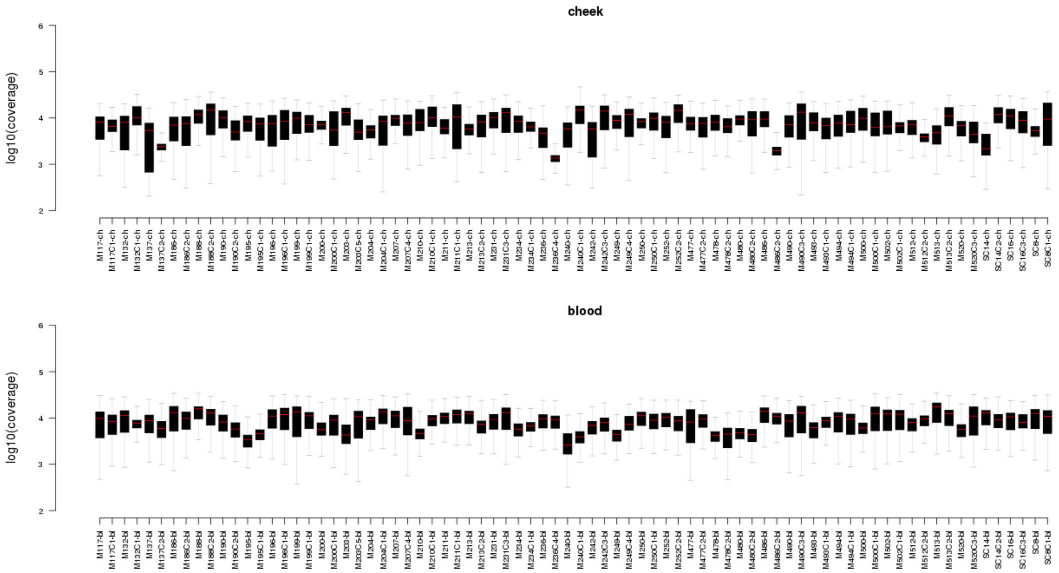

2.7 Define high quality heteroplasmic sites

We define high quality (HQ) sites as:

1. minor allele frequency (maf) $\geq$ 1%
2. coverage $\geq$ 1000
3. maf balance (1% in forward and reverse strands)
4. no strand bias
5. outside "problematic sites":
    - mtDNA homopolymeres
    - around the artificial "N" at position 3107
    - within 50 bp of the long range PCR primers

In [25]:
```
# Filter sites on minor allele frequncy (maf), coverage (cvrg) and wheather
# the sites are located in problematic regions
# The list of problematic region (maskRegions) is defined in cell 12 above
hq_sites = df[(df.maf>=0.01) & (df.cvrg>=1000) & ~df.position.isin(maskRegions)]
```

In [26]:
```
len(hq_sites)
```

Out[26]:
```
559
```

By applying these initial filters, we reduced the dataframe from ~2 million lines to **572** lines only, which is much more manageable. Next, we calculate strand bias and maf balance for these 572 sites. The strand bias calciulation is perfomed according to Guo Y et al. 2012

In [27]:
```
# Compute strand and minor allele frequency bias

def strand_stats(x, mafThreshold=0.01):
    falleles = ['A','C','G','T']
    ralleles = ['a','c','g','t']
    sample,position,major,minor,coverage,maf = x[['sample','position','major','minor','coverag
    fcounts = x[falleles]
    rcounts = x[ralleles]
    if minor!='.':
        index_major = falleles.index(major)
        index_minor = falleles.index(minor)

        fcount_minor = float(fcounts[index_minor])
        ftotal = fcount_minor + fcounts[index_major]

        rcount_minor = float(rcounts[index_minor])
        rtotal = rcount_minor + rcounts[index_major]

        minor_total = float(fcount_minor + rcount_minor)
        site_total = ftotal + rtotal

        try:
            strandBias = abs( (fcount_minor/ftotal) - (rcount_minor/rtotal) ) / (minor_total/s
        except:
            strandBias = np.nan

        try:
            maf_frwd = fcount_minor/sum(fcounts)
        except:
```

```
                maf_frwd = np.nan
            try:
                maf_rvrs = rcount_minor/sum(rcounts)
            except:
                maf_rvrs = np.nan

            if (maf_frwd>=mafThreshold) and (maf_rvrs>=mafThreshold):
                mafBalance = 1
            else:
                mafBalance = 0
        else:
            strandBias = float(2)
            mafBalance = 0

        return pd.Series([strandBias,mafBalance])
```

In [28]:
```
# Apply strand calculations to the data

biasCols = hq_sites.apply(strand_stats,axis=1,args=(0.01,))
biasCols.columns = ["strandBias","mafBalance"]
hq_sites = pd.concat([hq_sites,biasCols],axis=1)
```

In [29]:
```
# Filter on strand and maf balance

hq_sites = hq_sites[(hq_sites.strandBias<=1) & (hq_sites.mafBalance==1) ]
len(hq_sites)
```

Out[29]: 190

In [30]:
```
# Set bad sites column names
knownBadhqSites.columns=["sample","position"]

# Adjust naming convention for the anomalous grandmother-mother pair
knownBadhqSites.replace(to_replace=old,value=new,inplace=True)

# Transform bad sites into a hashable object
bad = set(knownBadhqSites.itertuples(index=False))

# Get a boolean array to filter high quality sites
good = [x not in bad for x in hq_sites[['sample','position']].itertuples(index=False)]

# Finally, filter high quality sites
hq_sites = hq_sites[good]
```

In [31]:
```
len(hq_sites)
#hq_sites.to_csv("hq173.txt",sep="\t",index=False)
```

Out[31]: 181

2.8 Test statistical significance of high quality sites

Finally, we calculate the significance of the minor allele frequency of a site provided the error rate at that position. The error rate is estimated from the remaining 155 samples, and the expected allele acounts are compared to the observed allele counts:

In [32]:
```
from scipy.stats import poisson
```

```
In [33]: # We define a poisson function that will take a single high quality site, and explore the vari
         # the position among the remaining samples


         def poisson_pval(current_df,sample):
             alleles = ['A','C','G','T','a','c','g','t']

             sample_counts = list(current_df.loc[current_df['sample']==sample, alleles].iloc[0,:])
             others_counts = list(current_df.loc[current_df['sample']!=sample, alleles].apply(sum,axis=
             sample_coverage = sum(sample_counts)

             observed_error = (sum(others_counts) - max(others_counts))/float(sum(others_counts))
             sample_nonMajor_counts = int(sample_coverage - max(sample_counts))

             pvalue = poisson.pmf(sample_nonMajor_counts, observed_error*sample_coverage)

             return pvalue
```

```
In [34]: poisson_pvalues = []

         for sample,position in hq_sites[["sample","position"]].itertuples(index=False):
             poisson_pvalues.append(poisson_pval(df[df['position']==position],sample))


         hq_sites["poisson"] = poisson_pvalues
         hq_sites = hq_sites[hq_sites.poisson<=0.05]
         len(hq_sites)
```

```
Out[34]: 181
```

As described in the paper, all sites were statiscally significant under the Poisson and Likelihood (not shown here) frameworks.

3 Screening for contamination

In our previous publication, Dickins, Rebolledo-Jaramillo, et al (2014) Controlling for contamination in resequencing studies with a reproducible web-based phylogentic approach BioTechniques, 56(3):134–141, we described warning signs of a potential contamination. They include: 1. Excess heteroplasmic sites (≥5 per sample) 2. Tight minor allele frequency distribution 3. Non-family related positions of heteroplasmic sites

We routinely apply our contamination detection pipeline, so we are confident our sites in the PNAS paper were not artifacts. As an example of the screening for contamination, we can plot the number of sites and the minor allele frequency distribution of all samples in the high quality sites set:

```
In [35]: # Make R aware of the hq_sites dataframe
         %R -i hq_sites
```

```
In [36]: %%R

         # Adjust value types in the hq_sites dataframe

         tonumeric = c(3:13,16:18)
         hq_sites[,tonumeric] = apply(hq_sites[,tonumeric], 2, function(x) as.numeric(as.character(x)))
         head(hq_sites,2)
```

```
        sample reference position    A   C  G    T    a   c  g    t cvrg
49743 M117C1-ch      chrM      214 1234   0  22    0 1581   0 36    0 2873
80535   M132-bl      chrM    14461    0 195  0 4356    0 183  0 4620 9354
      nalleles major minor     maf      sb strandBias mafBalance      poisson
49743        2     A     G 0.02019 0.23517  0.2351663          1 1.109776e-02
80535        2     T     C 0.04041 0.11746  0.1174580          1 4.761880e-06
```

```
In [37]: %%R -w 10 -h 15 -u in -r 72

         # Plot hq_sites number of sites and minor allele frequency distribution

         par(mar=c(4,15,4,0))
         boxplot(
           maf~sample,
           data=hq_sites,
           pch=16,cex=0.75,
           outcol="azure3",outline=T,
           whisklty=1,whiskwd=1.5,
           staplelwd=1.5,
           boxwex=0.75,boxcol="white",boxfill="cornflowerblue",
           horizontal=T,cex.axis=0.75,las=2,
           frame=F,xaxt="n",ylim=c(-0.01,0.5)
           )

         axis(1,at=seq(0,5,0.5)/10,lab=100*(seq(0,5,0.5)/10),cex.axis=0.75)
         title(xlab="minor allele frequency (%)",line=2.5,cex.axis=0.75)

         nsites = table(hq_sites[["sample"]])
         for (i in 1:length(nsites)){
           text(-0.01,i,lab=nsites[i],cex=0.75)
         }
         mtext("Nsites:",side=3,line=-2,at=0,adj=1.25)
```

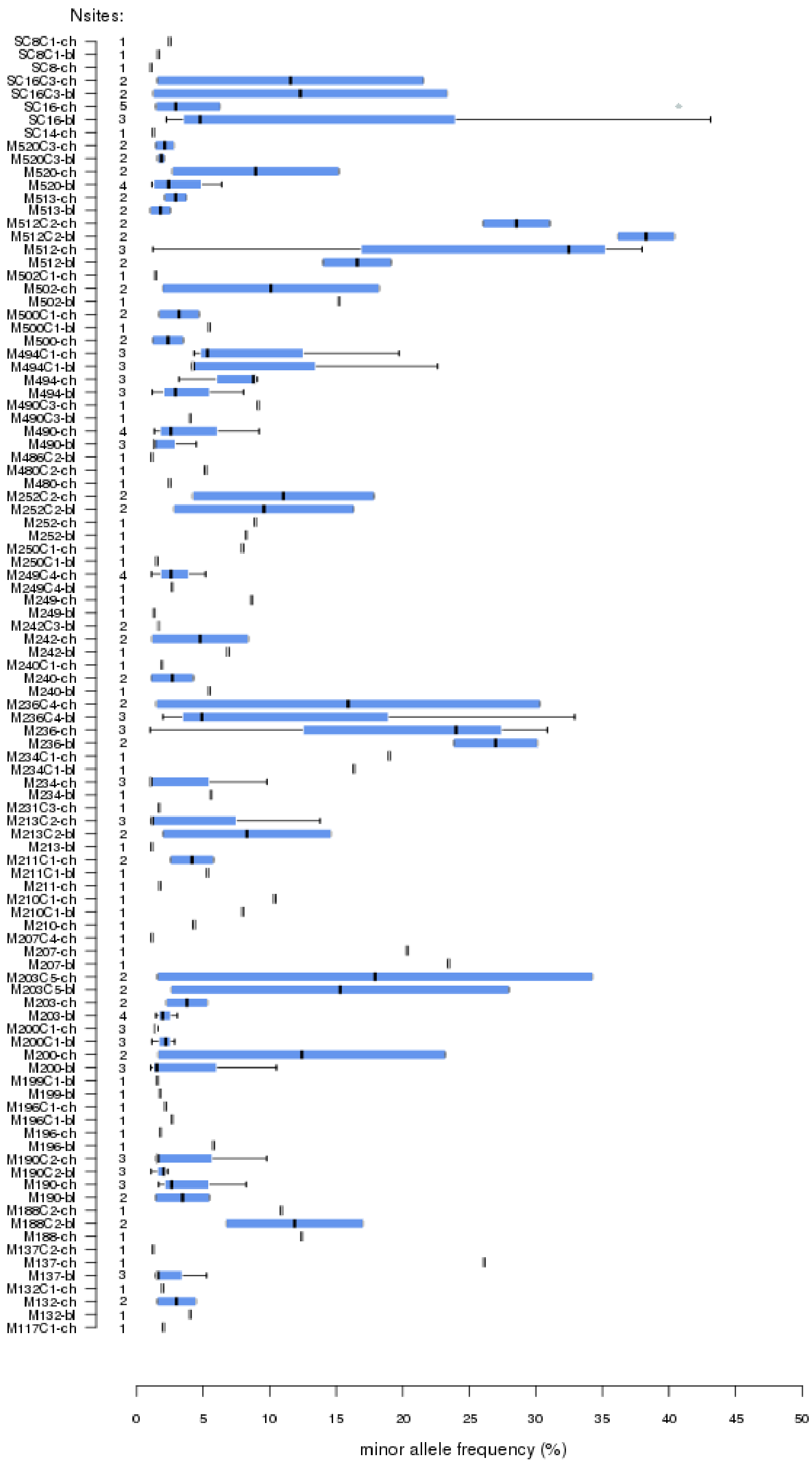
Nsites:
SC8C1-ch 1
SC8C1-bl 1
SC8-ch 1
SC16C3-ch 2
SC16C3-bl 2
SC16-ch 5
SC16-bl 3
SC14-ch 1
M520C3-ch 2
M520C3-bl 2
M520-ch 2
M520-bl 4
M513-ch 2
M513-bl 2
M512C2-ch 2
M512C2-bl 2
M512-ch 3
M512-bl 2
M502C1-ch 1
M502-ch 2
M502-bl 1
M500C1-ch 2
M500C1-bl 1
M500-ch 2
M494C1-ch 3
M494C1-bl 3
M494-ch 3
M494-bl 3
M490C3-ch 1
M490C3-bl 1
M490-ch 4
M490-bl 3
M486C2-bl 1
M480C2-ch 1
M480-ch 1
M252C2-ch 2
M252C2-bl 2
M252-ch 1
M252-bl 1
M250C1-ch 1
M250C1-bl 1
M249C4-ch 4
M249C4-bl 1
M249-ch 1
M249-bl 1
M242C3-bl 2
M242-ch 2
M242-bl 1
M240C1-ch 1
M240-ch 2
M240-bl 1
M236C4-ch 2
M236C4-bl 3
M236-ch 3
M236-bl 2
M234C1-ch 1
M234C1-bl 1
M234-ch 3
M234-bl 1
M231C3-ch 1
M213C2-ch 3
M213C2-bl 2
M213-bl 1
M211C1-ch 2
M211C1-bl 1
M211-ch 1
M210C1-ch 1
M210C1-bl 1
M210-ch 1
M207C4-ch 1
M207-ch 1
M207-bl 1
M203C5-ch 2
M203C5-bl 2
M203-ch 2
M203-bl 4
M200C1-ch 3
M200C1-bl 3
M200-ch 2
M200-bl 3
M199C1-bl 1
M199-bl 1
M196C1-ch 1
M196C1-bl 1
M196-ch 1
M196-bl 1
M190C2-ch 3
M190C2-bl 3
M190-ch 3
M190-bl 2
M188C2-ch 1
M188C2-bl 2
M188-ch 1
M137C2-ch 1
M137-ch 1
M137-bl 3
M132C1-ch 1
M132-ch 2
M132-bl 1
M117C1-ch 1
0 5 10 15 20 25 30 35 40 45 50
minor allele frequency (%)

3.1 Placing high quality sites into *quartets*

For each high quality site, we can retrieve the minor allele frequency information for the remaining 3 samples in the family collection. So, a quartet is simply a tabulation of the minor allele frequency for the mother blood and cheek, and her child blood and cheek, for the same site. Below is an example of a quarted for family **M494**:

family	position	major	minor	mother_cheek	mother_blood	child_cheek	child_blood
M494	9196	G	A	0.032	0.030	0.000	0.000

However, before we can do that, we need to add family information to the high quality sites. We will do that by extracting the family id from each sample's id. It is also useful to have a way to split the data by tissue or member of the pair, so we will add the columns `family`, `tissue` and `member`, accordingly.

```
In [38]: # Get family id from sample id
         # i.e. M512C1-ch returns M512

         def getfamlabels(samplename):
             nameparts = ["".join(x) for _, x in itertools.groupby(samplename, key=str.isdigit)]
             family = "".join(nameparts[:2])
             if "-ch" in nameparts:
                 tissue = "cheek"
             else:
                 tissue = "blood"

             if len(nameparts)>3:
                 pairclass = "child"
             else:
                 pairclass = "mother"

             return pd.Series([family,tissue,pairclass])
```

```
In [39]: # Apply the above function to the data

         hq_sites[["family","tissue","member"]] = hq_sites["sample"].apply(getfamlabels)
```

```
In [40]: hq_sites.head(2)
```

```
Out[40]:           sample reference  position     A    C   G     T     a    c   g  \
         49743  M117C1-ch      chrM       214  1234    0  22     0  1581    0  36
         80535    M132-bl      chrM     14461     0  195   0  4356     0  183   0

                ...    major  minor      maf       sb strandBias  mafBalance  \
         49743  ...        A      G  0.02019  0.23517   0.235166         1.0
         80535  ...        T      C  0.04041  0.11746   0.117458         1.0

                poisson  family  tissue  member
         49743  0.011098    M117   cheek   child
         80535  0.000005    M132   blood  mother

         [2 rows x 23 columns]
```

In [41]:
```
# Now we can extract the unique quartets by selecting the "family" and "position" columns

unique_quartets = hq_sites[["family","position"]].drop_duplicates()
len(unique_quartets)
```

Out[41]: 108

In [42]:
```
unique_quartets.head(2)
```

Out[42]:

	family	position
49743	M117	214
80535	M132	14461

In [43]:
```
# For each family_id/position combination,
# retrieve the information for all 4 members of the quartets
# from the original data dataframe

def getQuartets(hqsite):
    position = hqsite['position']
    familyid = hqsite['family']
    pos_data = df[df['position'] == position]
    allmembers = [s for s in pos_data['sample'].drop_duplicates() if  s.startswith(familyid) ]
    mother = min([len(x) for x in allmembers])
    child = max([len(x) for x in allmembers])
    if len(allmembers) == 4:

        for member in allmembers:
            if len(member)==mother and member.endswith("-ch"):
                motherCheek = df[(df['sample']==member) & (df['position']==position)][['major'

            elif len(member)==mother and member.endswith("-bl"):
                motherBlood = df[(df['sample']==member) & (df['position']==position)][['major'

            elif len(member)==child and member.endswith("-ch"):
                childCheek = df[(df['sample']==member) & (df['position']==position)][['major',

            else:
                childBlood = df[(df['sample']==member) & (df['position']==position)][['major',

        return pd.Series([familyid,position]+list(motherCheek)+list(motherBlood)+list(childChe
    else:
        pass
```

In [44]:
```
# Apply getQuartets to data

quartets = unique_quartets.apply(getQuartets,axis=1)
```

In [45]:
```
# The following is necssary to remove empty rows from the dataframe
quartets = quartets.dropna()
```

In [46]:
```
quartets
```

Out[46]:

	0	1	2	3	4	5	6	7	8	9	10	11	12	\
49743	M117	214.0	A	G	0.00181	A	G	0.00043	A	G	0.02019	A	G	
80535	M132	14461.0	T	C	0.04461	T	C	0.04041	T	C	0.00145	T	C	
82828	M132	185.0	A	G	0.01535	A	G	0.00569	A	G	0.00522	A	G	

115798	M132	64.0	C	T	0.00307	C	T	0.00030	C	T	0.01945	C	T
140360	M137	8953.0	A	.	0.00000	A	G	0.01451	A	T	0.00037	A	.
145325	M137	13918.0	T	C	0.00105	T	C	0.01591	T	.	0.00000	T	.
147727	M137	16320.0	C	T	0.26120	C	T	0.05245	C	.	0.00000	C	T
191703	M137	11054.0	C	T	0.00018	C	.	0.00000	C	T	0.01242	C	T
308891	M188	16240.0	A	G	0.00103	A	G	0.00007	A	G	0.10872	A	G
328908	M190	3202.0	T	C	0.01641	T	C	0.01420	T	C	0.00043	T	C
330811	M190	5105.0	T	C	0.02625	T	C	0.05490	T	.	0.00000	T	.
342450	M190	215.0	A	G	0.08234	A	G	0.00097	A	G	0.00122	A	.
365134	M190	6379.0	T	.	0.00000	T	C	0.00056	T	C	0.01585	T	C
366061	M190	7306.0	T	C	0.00032	T	C	0.00049	T	C	0.01473	T	C
375236	M190	16482.0	A	G	0.00153	A	G	0.00033	A	G	0.09767	A	G
474020	M196	16172.0	T	C	0.01780	T	C	0.05783	T	C	0.00048	T	C
507120	M196	16274.0	G	A	0.00021	G	A	0.00038	G	A	0.02176	G	A
539922	M199	16150.0	C	T	0.00227	C	T	0.01761	C	T	0.00020	C	T
558655	M199	1747.0	G	T	0.00013	G	A	0.00035	G	A	0.00015	G	A
589762	M200	596.0	T	C	0.23206	T	C	0.10507	T	C	0.00340	T	C
597746	M200	8584.0	G	A	0.00541	G	A	0.01516	G	A	0.00064	G	.
602733	M200	13571.0	C	T	0.01600	C	T	0.01060	C	.	0.00000	C	T
623866	M200	1598.0	G	A	0.00124	G	A	0.00062	G	A	0.00334	G	A
631050	M200	8784.0	A	G	0.00046	A	G	0.00161	A	G	0.01430	A	G
634146	M200	11881.0	C	T	0.01271	C	T	0.01057	C	T	0.01582	C	T
649833	M200	11012.0	T	C	0.00145	T	C	0.00075	T	C	0.01424	T	C
666023	M203	11825.0	G	A	0.05327	G	A	0.03067	G	A	0.00024	G	A
666180	M203	11982.0	T	C	0.00133	T	C	0.01451	T	C	0.00212	T	C
666832	M203	12634.0	A	G	0.00106	A	G	0.02005	A	G	0.00152	A	G
666887	M203	12689.0	T	C	0.00024	T	C	0.01907	T	.	0.00000	T	C
...	...	...	..	..	...	..	..	...	..	..	...	..	..
1975402	M494	11635.0	C	T	0.09062	C	T	0.08026	C	T	0.19712	C	T
2000080	M494	3183.0	T	C	0.00290	T	C	0.00177	T	C	0.04316	T	C
2012839	M494	15948.0	A	G	0.00127	A	G	0.00058	A	G	0.05317	A	G
2046767	M500	204.0	T	C	0.01199	T	C	0.00118	T	.	0.00000	T	C
2046777	M500	214.0	A	G	0.03514	A	G	0.00148	A	G	0.00049	A	G
2067319	M500	4191.0	A	G	0.00236	A	G	0.00149	A	T	0.04714	A	T
2090706	M500	11043.0	A	G	0.00111	A	G	0.00111	A	G	0.01670	A	G
2113750	M502	2706.0	A	G	0.00067	A	G	0.00037	A	G	0.01431	A	G
2156362	M502	12193.0	A	G	0.01937	A	G	0.00108	A	G	0.00148	A	G
2163961	M512	3243.0	A	G	0.32476	A	G	0.13995	G	A	0.31067	G	A
2166255	M512	5539.0	G	A	0.37990	G	A	0.19155	A	G	0.26009	A	G
2179847	M512	2581.0	A	G	0.01234	A	G	0.00043	A	C	0.00045	A	G
2228303	M513	1391.0	T	C	0.03773	T	C	0.02549	T	C	0.00151	T	C
2242245	M513	16235.0	G	A	0.00788	G	A	0.01025	G	A	0.00564	G	A
2245112	M513	2581.0	A	G	0.02104	A	G	0.00043	A	G	0.00114	A	G
2291624	M520	200.0	G	A	0.02678	G	A	0.01453	G	A	0.00431	G	A
2293155	M520	1778.0	T	C	0.00875	T	C	0.03364	T	C	0.00062	T	C
2298590	M520	7221.0	T	C	0.00309	T	C	0.01158	T	.	0.00000	T	C
2307452	M520	16093.0	T	C	0.15206	C	T	0.06392	C	T	0.02813	C	T
2326004	M520	1555.0	A	G	0.00272	A	G	0.00172	A	G	0.01419	A	G
2329629	M520	5181.0	A	G	0.00020	A	G	0.00060	A	G	0.01118	A	G
2382193	SC8	9116.0	T	C	0.01087	T	C	0.00843	T	C	0.00030	T	A
2402442	SC8	13708.0	G	A	0.00058	G	A	0.00025	G	A	0.02488	G	A
2444730	SC14	6683.0	T	C	0.01268	T	C	0.00060	T	C	0.00088	T	C
2488965	SC16	2352.0	C	T	0.40731	C	T	0.43130	C	T	0.21583	C	T
2497760	SC16	11149.0	G	A	0.02945	G	A	0.02249	G	T	0.00015	G	A

2502781	SC16	16170.0	A	G	0.06300	A	G	0.04775	A	G	0.00031	A	.
2503365	SC16	185.0	A	G	0.01450	A	G	0.00908	A	G	0.00182	A	G
2512743	SC16	9565.0	G	A	0.01445	G	A	0.01051	G	A	0.00095	G	A
2519899	SC16	152.0	T	C	0.00178	T	C	0.00059	T	C	0.01532	T	C

	13
49743	0.00215
80535	0.00089
82828	0.00506
115798	0.00019
140360	0.00000
145325	0.00000
147727	0.00036
191703	0.00599
308891	0.06739
328908	0.00013
330811	0.00000
342450	0.00000
365134	0.02330
366061	0.01087
375236	0.02052
474020	0.00044
507120	0.02656
539922	0.00050
558655	0.01555
589762	0.00158
597746	0.00000
602733	0.00049
623866	0.01150
631050	0.02196
634146	0.02859
649833	0.00044
666023	0.00037
666180	0.00183
666832	0.00062
666887	0.00043
...	...
1975402	0.22615
2000080	0.04190
2012839	0.04255
2046767	0.00127
2046777	0.00027
2067319	0.05447
2090706	0.00028
2113750	0.00053
2156362	0.00141
2163961	0.40417
2166255	0.36141
2179847	0.00040
2228303	0.00055
2242245	0.00451
2245112	0.00044
2291624	0.00117
2293155	0.00047

```
2298590  0.00126
2307452  0.00672
2326004  0.01572
2329629  0.02127
2382193  0.00017
2402442  0.01640
2444730  0.00049
2488965  0.23353
2497760  0.00025
2502781  0.00000
2503365  0.00053
2512743  0.00046
2519899  0.01251

[105 rows x 14 columns]
```

In [47]:
```
# Set column names
# mc: mother cheek
# mb: mother blood
# cc: child cheek
# cb: child blood

quartets.columns = ["family","position","mcMajor","mcMinor","mcMAF","mbMajor","mbMinor","mbMAF
                    "ccMajor","ccMinor","ccMAF","cbMajor","cbMinor","cbMAF"]
```

In [48]:
```
quartets.head(2)
```

Out[48]:
```
      family  position mcMajor mcMinor    mcMAF mbMajor mbMinor    mbMAF  \
49743   M117     214.0       A       G  0.00181       A       G  0.00043
80535   M132   14461.0       T       C  0.04461       T       C  0.04041

      ccMajor ccMinor    ccMAF cbMajor cbMinor    cbMAF
49743       A       G  0.02019       A       G  0.00215
80535       T       C  0.00145       T       C  0.00089
```

We can add even more information to the quartets table. For instance, the impact of the alternative allele and the nucleotide change class:

In [49]:
```
# Define function for generating protein translation

def translate(sequence,gene):
    if len(str(sequence))%3!=0:
        add=3 - (len(str(sequence))%3)
    else:
        add=0

    if genedb[gene]["strand"]==1:
        modseq=str(sequence)+add*'A'
    else:
        modseq=str(sequence.reverse_complement())+add*'A'

    try:
        translation=str(Seq(modseq,IUPAC.unambiguous_dna).translate(table=2,cds=True))
    except:
        translation=[]
```

```
        return translation
```

In [50]:
```
# Define function for estimating evolutionary impact
# The function determines if a heteroplamic site
# synonymous/non-synonomous and if the change is transitional or transversional

def evoImpact(quartet):
    try:
        het,major,minor = quartet
        pos  = int(het)-1
        gene = [g for g in genedb if genedb[g]['end']>=pos>=genedb[g]['start']][0]



        if gene in [feature.qualifiers['gene'][0] for feature in rCRS.features if feature.type=

            majorseq = rCRS.seq.tomutable()
            minorseq = rCRS.seq.tomutable()
            majorseq[pos] = major
            minorseq[pos] = minor
            ref_seq = rCRS.seq[genedb[gene]["start"]:genedb[gene]["end"]]
            major_seq = majorseq[genedb[gene]["start"]:genedb[gene]["end"]]
            minor_seq = minorseq[genedb[gene]["start"]:genedb[gene]["end"]]

            if (translate(ref_seq,gene)==translate(minor_seq,gene)):
                ptimpact = "syn"
            else:
                ptimpact = "nonsyn"
        else:
            ptimpact = "-"

        ntClass={'pu':['A','G'],'py':['C','T']}
        majorClass=[k for k,v in ntClass.iteritems() if major in v]
        minorClass=[k for k,v in ntClass.iteritems() if minor in v]
        if majorClass==minorClass:
            ntimpact='ts'
        else:
            ntimpact='tv'

        return pd.Series([ptimpact,genedb[gene]['class'],ntimpact])

    except:
        pass
```

In [51]:
```
# Using BioPython rCRS object defined in cell 10
# Parse mitochondrial genome features

genedb = dict()
labs = ["class","start","end","strand"]
for feature in rCRS.features:
    if feature.type in ["rRNA","tRNA","CDS"]:
        ftype = feature.type
```

```
            name = feature.qualifiers['gene'][0]
            start = int(feature.location.start)
            end = int(feature.location.end)
            strand = int(feature.location.strand)
            genedb[name] = dict(zip(labs,[ftype,start,end,strand]))
    genedb['D-loop1'] = dict(zip(labs,["Dloop",0,576,1]))
    genedb['D-loop2'] = dict(zip(labs,["Dloop",16023,16569,1]))
```

In [52]:
```
a = quartets.loc[282115:328908]
a.head(20)
#a[['position','mbMajor','mbMinor']].apply(evoImpact,axis=1)
```

Out[52]:
```
        family  position mcMajor mcMinor    mcMAF mbMajor mbMinor    mbMAF  \
308891    M188   16240.0       A       G  0.00103       A       G  0.00007
328908    M190    3202.0       T       C  0.01641       T       C  0.01420

       ccMajor ccMinor    ccMAF cbMajor cbMinor    cbMAF
308891       A       G  0.10872       A       G  0.06739
328908       T       C  0.00043       T       C  0.00013
```

In [53]:
```
# We set the ancestral state to the alleles found in the mother's blood sample.

quartets[["ptchange","class","ntchange"]] = quartets[['position','mbMajor','mbMinor']].apply(e
```

Finalized quartets table:

In [54]:
```
quartets.head(2)
```

Out[54]:
```
       family  position mcMajor mcMinor    mcMAF mbMajor mbMinor    mbMAF  \
49743    M117     214.0       A       G  0.00181       A       G  0.00043
80535    M132   14461.0       T       C  0.04461       T       C  0.04041

      ccMajor ccMinor    ccMAF cbMajor cbMinor    cbMAF ptchange  class  \
49743       A       G  0.02019       A       G  0.00215        -  Dloop
80535       T       C  0.00145       T       C  0.00089   nonsyn    CDS

      ntchange
49743       ts
80535       ts
```

3.2 Plot the number of heteroplasmic sites per individual or family (Fig. S11)

In [55]:
```
# Since we modified the hq_sites dataframe, we have to reload it in R

%R -i hq_sites,quartets
```

In [56]:
```
%%R

# Adjust value types in the hq_sites dataframe
tonumeric = c(3:13,16:18)
hq_sites[,tonumeric] = apply(hq_sites[,tonumeric], 2, function(x) as.numeric(as.character(x)))
head(hq_sites,2)
```

```
       sample reference position    A   C  G    T    a   c  g    t cvrg
49743 M117C1-ch      chrM      214 1234   0 22    0 1581   0 36    0 2873
80535   M132-bl      chrM    14461    0 195   0 4356    0 183   0 4620 9354
```

```
      nalleles major minor     maf      sb strandBias mafBalance      poisson
49743        2     A     G 0.02019 0.23517  0.2351663          1 1.109776e-02
80535        2     T     C 0.04041 0.11746  0.1174580          1 4.761880e-06
      family tissue member
49743   M117  cheek  child
80535   M132  blood mother
```

In [57]: %%R

```
# Frequency (number of sites per individual)

getFreq = function(data,tissue,member) {

    siteFreq = data.frame(table(table(as.character(data[(data[["tissue"]]==tissue) & (data[["me
    siteFreq = unlist(apply(siteFreq,1,FUN=function(x) rep(x[1],x[2])))
    siteFreq = as.numeric(c(rep(0,39-length(siteFreq)),siteFreq))

    return(siteFreq)
        }
```

In [58]: %%R

```
# Size of circles

symbolPlot = function(data,pos) {

    symbols(rep(pos,length(unique(data))),
            sort(unique(data)),circles=(data.frame(table(data))$Freq)*0.01,
            add=T,inches=F,bg="black")
}
```

In [59]: %%R

```
# Backbone boxplot

boxPlotNsites = function(data,pos,addOpt="False"){
    boxplot(data,ylim=c(-2,maxSites),frame=F,axes=F,xlim=c(1,7),at=pos,col=rgb(0,0,0,0),
            boxlwd=2,boxcol="coral3",medcol="coral3",whisklty="solid",whiskcol="coral3",
            staplecol="coral3",add=as.logical(addOpt),outline=F)

}
```

In [60]: %%R -w 11 -h 8 -u in -r 72

```
mc = getFreq(hq_sites,"cheek","mother")
mb = getFreq(hq_sites,"blood","mother")
cc = getFreq(hq_sites,"cheek","child")
cb = getFreq(hq_sites,"blood","child")

fam = data.frame(table(table(quartets[["family"]])))
fam = unlist(apply(fam,1,FUN=function(x) rep(as.numeric(x[1]),x[2])))
fam = as.numeric(c(rep(0,39-length(fam)),fam))
```

```
maxSites = max(c(mc,mb,cc,cb,fam))
par(mar=c(2,2,2,1))
par(oma=c(0,0,0,0))

plot(1:7,1:7,type="n",ylim=c(-2,maxSites),frame=F,axes=F)

symbolPlot(mc,2)
symbolPlot(mb,3)
symbolPlot(cc,4)
symbolPlot(cb,5)
symbolPlot(fam,6)

Lab = c("Maternal \ncheek","Maternal \nblood","Child \ncheek","Child \nblood","Family")

axis(1,at=2:6,lab=Lab,pos=-1.5,las=1,cex.axis=0.8,tck=-0.01)
axis(2,at=0:maxSites,lab=0:maxSites,pos=1,las=2,cex.axis=0.8)
mtext("Number of point heteroplasmies",2,1,cex=0.8,adj=0.7)


par(new=T)
boxPlotNsites(mc,2)
boxPlotNsites(mb,3,"True")
boxPlotNsites(cc,4,"True")
boxPlotNsites(cb,5,"True")
boxPlotNsites(fam,6,"True")
```

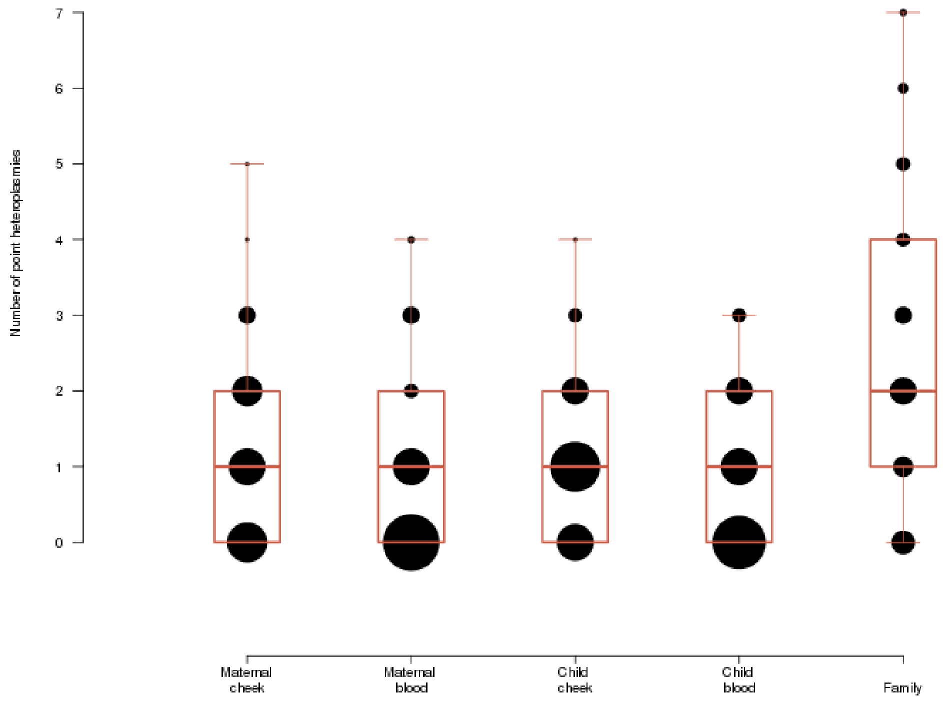

3.3 Plot distribution of high quality heteroplasmies (Fig. S10)

In [61]: %%R

```
data = quartets
uniqueFamilies = sort(unique(as.character(data$family)))
faid = cbind(1:length(uniqueFamilies),uniqueFamilies)

plotid=c()
for (s in as.character(data$family)){
    plotid=c(plotid,faid[faid[,2]==s,1])
}
data$id = as.numeric(plotid)
data$position = as.numeric(as.character(data$position))
head(data)
```

```
       family position mcMajor mcMinor   mcMAF mbMajor mbMinor   mbMAF ccMajor
49743    M117      214       A       G 0.00181       A       G 0.00043       A
80535    M132    14461       T       C 0.04461       T       C 0.04041       T
82828    M132      185       A       G 0.01535       A       G 0.00569       A
115798   M132       64       C       T 0.00307       C       T 0.00030       C
140360   M137     8953       A       . 0.00000       A       G 0.01451       A
145325   M137    13918       T       C 0.00105       T       C 0.01591       T
       ccMinor   ccMAF cbMajor cbMinor   cbMAF ptchange class ntchange id
49743        G 0.02019       A       G 0.00215        - Dloop       ts  1
80535        C 0.00145       T       C 0.00089   nonsyn   CDS       ts  2
82828        G 0.00522       A       G 0.00506        - Dloop       ts  2
115798       T 0.01945       C       T 0.00019        - Dloop       ts  2
140360       T 0.00037       A       . 0.00000   nonsyn   CDS       ts  3
145325       . 0.00000       T       . 0.00000   nonsyn   CDS       ts  3
```

In [62]: %%R

```
# Define colors for each mitochondrial genome features

alp  =200
trna ="blue"
rrna ="lightseagreen"
prot ="orange"
dloop="red"

colors=c()
for (c in data[["class"]]) if (c=="Dloop") {
    colors=c(colors,dloop)
    } else if (c=="tRNA") {
        colors=c(colors,trna)
    } else if (c=="rRNA") {
        colors=c(colors,rrna)
    } else {
    colors=c(colors,prot)
```

```
}
data$colors=colors
```

In [63]: %%R

```
# Define symbols depending on whether a site is syn/nonsyn or ts/tv

symbol=c()
for (i in 1:nrow(data)){
    if (data[["ntchange"]][i]=="tv" & data[["ptchange"]][i]=="nonsyn") {symbol=c(symbol,17)
    } else if (data[["ntchange"]][i]=="tv" & data[["ptchange"]][i]!="nonsyn") {symbol=c(symbol
    } else if (data[["ptchange"]][i]=="syn") {
          symbol=c(symbol,16)
    } else {
          symbol=c(symbol,1)}
    }
data$symbol=symbol
```

In [64]: %%R -w 5 -h 4 -u in -r 144

```
# Plot area
par(mar=c(2,2,2,1))
par(oma=c(0,0,0,0))
par(las=2)
plot(data$position,data$id*5+5,xlim=c(1,16569), ylim=c(-80,170),frame="False",ylab="",axes=F, 

# Grid
for (i in data$id) {segments(1,i*5+5,16569,i*5+5,col="grey90")}
for (i in c(1,seq(500,16500,500))) {segments(i,5,i,162,col="grey90")}
for (i in seq(500,16500,1000)) {text(i,163,lab=i,col="black",cex=0.25)}
mtext("Family",3,-1.2,las=1,adj=-0.07,cex=0.5)
mtext("coord:",3,-1.35,las=1,adj=0.01,cex=0.3)

# Actual plot
points(data$position,data$id*5+5,xlim=c(1,16569), ylim=c(-80,170),pch=data$symbol,ylab="",col=

# Axes
abline(h=5)
lab=unique(data[,c(1,18)])
axis(2,pos=-500,at=sort(unique(data$id*5+5)),labels=lab[order(lab$id),][,1],cex.axis=0.4)

# Legend
legend(1,180,legend=c("D-loop","tRNA","rRNA","cds-syn","cds-nonSyn","transversion"),
       fill=c(NA,NA,NA,NA,NA,NA),border=c(rep("white",4),NA,NA),pch=c(1,1,1,1,16,2),
       col=c(dloop,trna,rrna,prot,prot,"black"),cex=0.5,bty="n",pt.cex=0.7,horiz=T,
       x.intersp=c(0.7,0.7,0.7,0.7,0.7,1),text.width=1350)


# mtDNA genes

Arrows(1,0,576,0,arr.length=0.05,arr.type='simple')
text(576,0,labels='DLOOP',cex=0.5,pos=4)
Arrows(576,-5,647,-5,arr.length=0.05,arr.type='simple')
text(647,-5,labels='TRNF',cex=0.5,pos=4)
Arrows(647,-10,1601,-10,arr.length=0.05,arr.type='simple')
```

```
text(1601,-10,labels='RNR1',cex=0.5,pos=4)
Arrows(1601,-15,1670,-15,arr.length=0.05,arr.type='simple')
text(1670,-15,labels='TRNV',cex=0.5,pos=4)
Arrows(1670,-20,3229,-20,arr.length=0.05,arr.type='simple')
text(3229,-20,labels='RNR2',cex=0.5,pos=4)
Arrows(3229,-25,3304,-25,arr.length=0.05,arr.type='simple')
text(3304,-25,labels='TRNL1',cex=0.5,pos=4)
Arrows(3306,-30,4262,-30,arr.length=0.05,arr.type='simple')
text(4262,-30,labels='ND1',cex=0.5,pos=4)
Arrows(4262,-35,4331,-35,arr.length=0.05,arr.type='simple')
text(4331,-35,labels='TRNI',cex=0.5,pos=4)
Arrows(4328,-40,4400,-40,arr.length=0.05,code=1,arr.type='simple')
text(4400,-40,labels='TRNQ',cex=0.5,pos=4)
Arrows(4401,-45,4469,-45,arr.length=0.05,arr.type='simple')
text(4469,-45,labels='TRNM',cex=0.5,pos=4)
Arrows(4469,-50,5511,-50,arr.length=0.05,arr.type='simple')
text(5511,-50,labels='ND2',cex=0.5,pos=4)
Arrows(5511,-55,5579,-55,arr.length=0.05,arr.type='simple')
text(5579,-55,labels='TRNW',cex=0.5,pos=4)
Arrows(5586,-60,5655,-60,arr.length=0.05,code=1,arr.type='simple')
text(5655,-60,labels='TRNA',cex=0.5,pos=4)
Arrows(5656,-65,5729,-65,arr.length=0.05,code=1,arr.type='simple')
text(5729,-65,labels='TRNN',cex=0.5,pos=4)
Arrows(5760,-70,5826,-70,arr.length=0.05,code=1,arr.type='simple')
text(5826,-70,labels='TRNC',cex=0.5,pos=4)
Arrows(5825,-75,5891,-75,arr.length=0.05,code=1,arr.type='simple')
text(5891,-75,labels='TRNY',cex=0.5,pos=4)
Arrows(5903,-5,7445,-5,arr.length=0.05,arr.type='simple')
text(7445,-5,labels='COX1',cex=0.5,pos=4)
Arrows(7445,-10,7514,-10,arr.length=0.05,code=1,arr.type='simple')
text(7514,-10,labels='TRNS1',cex=0.5,pos=4)
Arrows(7517,-15,7585,-15,arr.length=0.05,arr.type='simple')
text(7585,-15,labels='TRND',cex=0.5,pos=4)
Arrows(7585,-20,8269,-20,arr.length=0.05,arr.type='simple')
text(8269,-20,labels='COX2',cex=0.5,pos=4)
Arrows(8294,-25,8364,-25,arr.length=0.05,arr.type='simple')
text(8364,-25,labels='TRNK',cex=0.5,pos=4)
Arrows(8365,-30,8572,-30,arr.length=0.05,arr.type='simple')
text(8572,-30,labels='ATP8',cex=0.5,pos=4)
Arrows(8526,-35,9207,-35,arr.length=0.05,arr.type='simple')
text(9207,-35,labels='ATP6',cex=0.5,pos=4)
Arrows(9206,-40,9990,-40,arr.length=0.05,arr.type='simple')
text(9990,-40,labels='COX3',cex=0.5,pos=4)
Arrows(9990,-45,10058,-45,arr.length=0.05,arr.type='simple')
text(10058,-45,labels='TRNG',cex=0.5,pos=4)
Arrows(10058,-50,10404,-50,arr.length=0.05,arr.type='simple')
text(10404,-50,labels='ND3',cex=0.5,pos=4)
Arrows(10404,-55,10469,-55,arr.length=0.05,arr.type='simple')
text(10469,-55,labels='TRNR',cex=0.5,pos=4)
Arrows(10469,-5,10766,-5,arr.length=0.05,arr.type='simple')
text(10766,-5,labels='ND4L',cex=0.5,pos=4)
Arrows(10759,-10,12137,-10,arr.length=0.05,arr.type='simple')
text(12137,-10,labels='ND4',cex=0.5,pos=4)
Arrows(12137,-15,12206,-15,arr.length=0.05,arr.type='simple')
```

```
text(12206,-15,labels='TRNH',cex=0.5,pos=4)
Arrows(12206,-20,12265,-20,arr.length=0.05,arr.type='simple')
text(12265,-20,labels='TRNS2',cex=0.5,pos=4)
Arrows(12265,-25,12336,-25,arr.length=0.05,arr.type='simple')
text(12336,-25,labels='TRNL2',cex=0.5,pos=4)
Arrows(12336,-30,14148,-30,arr.length=0.05,arr.type='simple')
text(14148,-30,labels='ND5',cex=0.5,pos=4)
Arrows(14148,-35,14673,-35,arr.length=0.05,code=1,arr.type='simple')
text(14673,-35,labels='ND6',cex=0.5,pos=4)
Arrows(14673,-40,14742,-40,arr.length=0.05,code=1,arr.type='simple')
text(14742,-40,labels='TRNE',cex=0.5,pos=4)
Arrows(14746,-45,15887,-45,arr.length=0.05,arr.type='simple')
text(15887,-45,labels='CYTB',cex=0.5,pos=4)
Arrows(15887,-50,15953,-50,arr.length=0.05,arr.type='simple')
text(15953,-50,labels='TRNT',cex=0.5,pos=4)
Arrows(15955,-55,16023,-55,arr.length=0.05,code=1,arr.type='simple')
text(16023,-55,labels='TRNP',cex=0.5,pos=4)
Arrows(16024,0,16569,0,arr.length=0.05,arr.type='simple')
text(16024,0,labels='DLOOP',cex=0.5,pos=2)
```

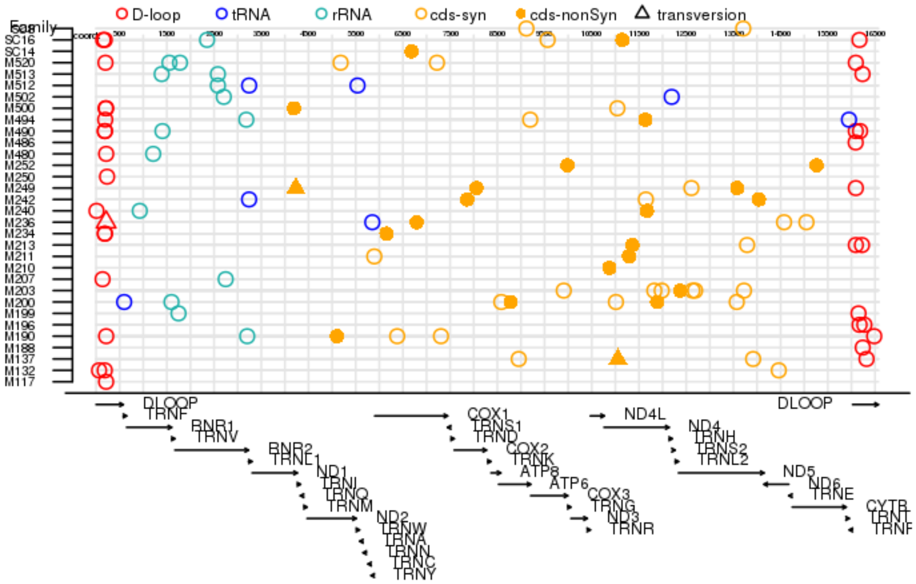

3.4 Plot correlations in minor allele frequencies (Fig. 1)

In [65]:
```
%%R
#head(quartets,3)
quartets[as.character(quartets$cbMinor)!=as.character(quartets$ccMinor),]
```

```
 family position mcMajor mcMinor   mcMAF mbMajor mbMinor   mbMAF ccMajor
```

```
140360    M137     8953       A       . 0.00000       A       G 0.01451       A
147727    M137    16320       C       T 0.26120       C       T 0.05245       C
342450    M190      215       A       G 0.08234       A       G 0.00097       A
597746    M200     8584       G       A 0.00541       G       A 0.01516       G
602733    M200    13571       C       T 0.01600       C       T 0.01060       C
666887    M203    12689       T       C 0.00024       T       C 0.01907       T
788952    M207     2746       T       C 0.20339       T       C 0.23444       T
1193442   M236    14573       A       G 0.30872       A       G 0.30129       A
1244595   M240      926       A       G 0.04277       A       G 0.05453       A
1302356   M240    11668       C       T 0.00028       C       T 0.00041       C
1718621   M480     1211       G       A 0.02484       G       A 0.01028       G
1833848   M490     1407       T       C 0.00705       T       C 0.01344       T
2046767   M500      204       T       C 0.01199       T       C 0.00118       T
2179847   M512     2581       A       G 0.01234       A       G 0.00043       A
2298590   M520     7221       T       C 0.00309       T       C 0.01158       T
2382193    SC8     9116       T       C 0.01087       T       C 0.00843       T
2497760   SC16    11149       G       A 0.02945       G       A 0.02249       G
2502781   SC16    16170       A       G 0.06300       A       G 0.04775       A
        ccMinor   ccMAF cbMajor cbMinor   cbMAF ptchange class ntchange
140360        T 0.00037       A       . 0.00000   nonsyn   CDS       ts
147727        . 0.00000       C       T 0.00036        - Dloop       ts
342450        G 0.00122       A       . 0.00000        - Dloop       ts
597746        A 0.00064       G       . 0.00000   nonsyn   CDS       ts
602733        . 0.00000       C       T 0.00049   nonsyn   CDS       ts
666887        . 0.00000       T       C 0.00043   nonsyn   CDS       ts
788952        A 0.00031       T       C 0.00012        -  rRNA       ts
1193442       . 0.00000       A       G 0.00026   nonsyn   CDS       ts
1244595       G 0.00034       A       . 0.00000        -  rRNA       ts
1302356       T 0.01888       C       . 0.00000      syn   CDS       ts
1718621       A 0.00007       G       . 0.00000        -  rRNA       ts
1833848       C 0.00034       T       . 0.00000        -  rRNA       ts
2046767       . 0.00000       T       C 0.00127        - Dloop       ts
2179847       C 0.00045       A       G 0.00040        -  rRNA       ts
2298590       . 0.00000       T       C 0.00126   nonsyn   CDS       ts
2382193       C 0.00030       T       A 0.00017   nonsyn   CDS       ts
2497760       T 0.00015       G       A 0.00025      syn   CDS       ts
2502781       G 0.00031       A       . 0.00000        - Dloop       ts
```

There are a few cases of reversal of mninor allele frequencies between two tissues of the same individual, or between a mother and her child. Consequently, it is necessary to fix the “ancestral” allele, and we arbitrarily decided to use the maternal blood as the ancestral state.

In [66]: %%R

```
# mb = maternal blood
# mc = maternal cheek
# cb = child blood
# cc = child cheek

adjustMAF = function(row){

    mbMajor = row[["mbMajor"]]
```

```
            mbMinor = row[["mbMinor"]]
            mcMajor = row[["mcMajor"]]
            mcMinor = row[["mcMinor"]]
            ccMajor = row[["ccMajor"]]
            ccMinor = row[["ccMinor"]]
            cbMajor = row[["cbMajor"]]
            cbMinor = row[["cbMinor"]]

            if ((c(mbMajor,mbMinor) == c(mcMinor,mcMajor)) & (mcMinor!=".")){
                mcMAFadj = 1 - as.numeric(row[["mcMAF"]])
            }else{
                mcMAFadj = as.numeric(row[["mcMAF"]])
            }


            if ((c(mbMajor,mbMinor) == c(ccMinor,ccMajor)) & (ccMinor!=".")){
                ccMAFadj = 1 - as.numeric(row[["ccMAF"]])
            }else{
                ccMAFadj = as.numeric(row[["ccMAF"]])
            }


            if ((c(mbMajor,mbMinor) == c(cbMinor,cbMajor)) & (cbMinor!=".")){
                cbMAFadj = 1 - as.numeric(row[["cbMAF"]])
            }else{
                cbMAFadj = as.numeric(row[["cbMAF"]])
            }

            return(c(mcMAFadj,ccMAFadj,cbMAFadj))
        }
```

In [67]: %%R

```
        adjustedMAF = data.frame(t(apply(quartets, 1, adjustMAF)))
        colnames(adjustedMAF) = c("mc","cc","cb")

        head(adjustedMAF,2)
```

```
           mc      cc      cb
49743 0.00181 0.02019 0.00215
80535 0.04461 0.00145 0.00089
```

Due to the adjustment of MAF based on the maternal state, comparing the child tissues independently of the mother's, require an additional adjustment.

In [68]: %%R

```
        ccx=c()
        for (maf in adjustedMAF$cc) {if (maf>0.5) ccx=c(ccx,(1-maf)) else ccx=c(ccx,maf)}
        cbx=c()
        for (maf in adjustedMAF$cb) {if (maf>0.5) cbx=c(cbx,(1-maf)) else cbx=c(cbx,maf)}
```

In [69]: %%R

```
xyplot = function(x,y,sub,xtissue,ytissue,case){

    xLab = paste("het. allele frequency (",xtissue,")",sep="")
    yLab = paste("het. allele frequency (",ytissue,")",sep="")

    plot(x,y,pch=20,col="#00000078",axes=F,xlab=xLab,
         ylab=yLab,cex.lab=0.85,cex=2,xlim=c(0,1),ylim=c(0,1))

    abline(lm(x~y), col="darkgrey",lwd=1)
    mylabel = bquote(italic(R)^2 == .(round(summary(lm(x~y))$r.squared,2)))

    text(-0.05,0.7, pos=4,labels=mylabel,font=2,cex=1)
    text(-0.05,0.9,labels=case,cex=1,pos=4)
    mtext(sub,3,0.5,at=0,cex=1,font=1)
    axis(1,at=c(0,0.5,1))
    axis(2,at=c(0,0.5,1))
}
```

```
In [70]: %%R -w 4 -h 4 -u in -r 144

par(mfrow=c(2,2))
par(oma=c(0,0,0,0))
par(mar=c(3,2.5,2,1))

par(mgp=c(1.5,0.25,0.25))
par(tck=-0.05)

xyplot(ccx,cbx,"A","cheek","blood","CHILD")
xyplot(adjustedMAF$mc,quartets$mbMAF,"B","cheek","blood","MOTHER")
xyplot(adjustedMAF$mc,ccx,"C","mother","child","CHEEK")
xyplot(quartets$mbMAF,cbx,"D","mother","child","BLOOD")
```

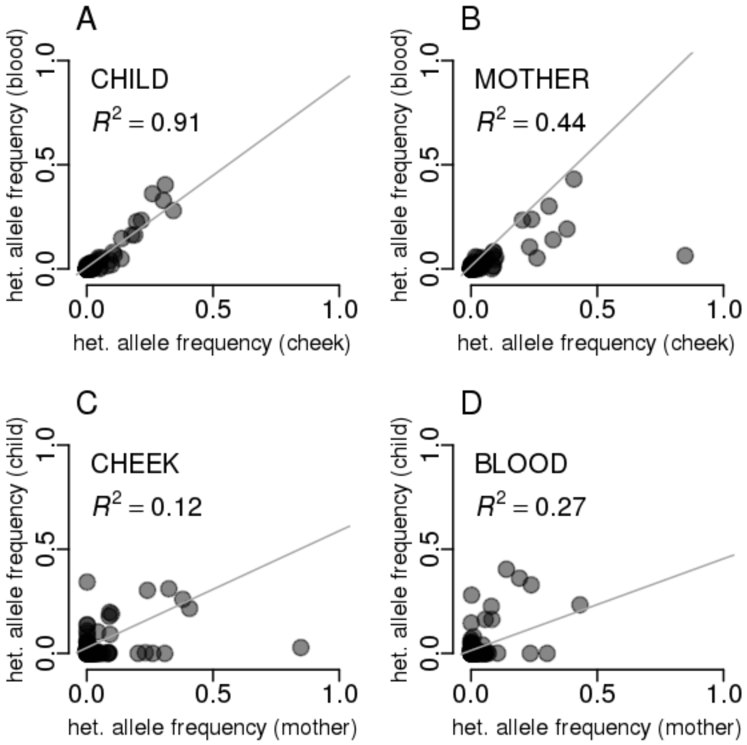

3.5 Plot the bottleneck size (Fig. S15)

In [71]: %%R

```
# We calculated the bottleneck size by comparing
# the allele frequency of the minor allele in the mother and her child

bottleneckData = data.frame(
    mc=adjustedMAF$mc,
    mb=quartets[["mbMAF"]],
    cc=adjustedMAF$cc,
    cb=adjustedMAF$cb)


# We used the average of the two tissues in an individual

bottleneckData[["meanM"]] = apply(bottleneckData[,1:2], 1,mean)
bottleneckData[["meanC"]] = apply(bottleneckData[,3:4], 1,mean)


# And modeled the bottleneck as in Millar et al, 2008

bottleneckData$bn1 = (bottleneckData$meanM*(1-bottleneckData$meanM))/(bottleneckData$meanC-bot
bottleneckData$bn1.cheek =(bottleneckData$mc*(1-bottleneckData$mc))/(bottleneckData$mc-bottlen
bottleneckData$bn1.blood =(bottleneckData$mb*(1-bottleneckData$mb))/(bottleneckData$mb-bottlen
```

```
# Select cases where there is evidence of the minor allele in the maternal linage (i.e. the mi
# both tissues of the mother, at least 1% in one of the tissues, and 0.2% in the other tissue)



bn1.m = bottleneckData[(bottleneckData$mc>=0.01|bottleneckData$mb>=0.01) &
                        (bottleneckData$mc>=0.002 & bottleneckData$mb>=0.002),][["bn1"]]

bn1.cheek = bottleneckData[bottleneckData$mc>=0.01,][["bn1.cheek"]]
bn1.blood = bottleneckData[bottleneckData$mb>=0.01,][["bn1.blood"]]
```

In [72]: %%R

```
length(bn1.m)
```

```
[1] 50
```

In [73]: %%R

```
# Accounting for mitotic segregation

mitotic = function(row){
    mc = row[1]
    mb = row[2]
    cc = row[3]
    cb = row[4]
    variance = ((mc-cc)^2+(mc-cb)^2+(mb-cc)^2+(mb-cc)^2-2*(mc-mb)^2-2*(cc-cb)^2)/4
    return(variance)
}

bottleneckData[["mitotvar"]] = apply(bottleneckData,1,mitotic)
bottleneckData$bn2 = (bottleneckData$meanM*(1-bottleneckData$meanM))/(bottleneckData$mitotvar)
bn2.m = bottleneckData[(bottleneckData$mc>=0.01|bottleneckData$mb>=0.01) &
                        (bottleneckData$mc>=0.002 & bottleneckData$mb>=0.002),][["bn2"]]
```

In [74]: %%R

```
# We removed negative or indetermined estimates of the bottleneck

bn2.m = bn2.m[bn2.m>0]
```

In [75]: %%R

```
length(bn2.m)
```

```
[1] 45
```

In [76]: %%R

```
bn1.m
```

```
 [1]    23.816855   359.631901     5.388898    66.758429    23.645718
 [6]    26.075053   107.006629     5.081444   102.508491    76.998633
[11]   103.069255    23.161960    66.648376     3.575002     5.999405
[16]  4576.759055    87.090388     7.145162   543.823984   681.303195
```

```
[21]     2.280578    30.658853    19.692368   286.394261    12.206464
[26]    20.436016    10.789702    56.171303    99.896336    68.929415
[31] 10199.573616    81.633597   162.572697    20.286884    31.950263
[36]     4.906696     1.059908     1.253351    32.734057   564.244306
[41]    63.027094    48.650744   161.959066     1.290042   107.814053
[46]     6.428074    38.090392    17.154591   103.400598    88.887083
```

In [77]:
```
%%R


# Get the actual stats before transforming the data
data_tmp=list(bn1.m,bn2.m)
medians=c()
first=c()
third=c()

for (i in 1:2){
    medians=c(medians,median(unlist(data_tmp[i])))
    first=c(first,summary(unlist(data_tmp[i]))[2])
    third=c(third,summary(unlist(data_tmp[i]))[5])
}

bn1.m = log10(bn1.m)
bn2.m = log10(bn2.m)
```

In [78]:
```
%%R -w 6 -h 5 -u in -r 144

par(oma=c(0,0,4,0))
par(bty="n")
par(xpd=TRUE)
par(lwd=1.5)
par(pch=20)


# blank boxplot
boxplot(bn1.m,xlim=c(0,5),ylim=c(-1,3),at=1,frame=F,axes=F,
        ylab="",medcol="white",whiskcol="white",boxcol="white",staplecol="white")

# actual drawings
vioplot(bn1.m,ylim=c(-1,3),at=1,col="royalblue",add=T,border=NA)
vioplot(bn2.m,ylim=c(-1,3),at=2,col="tomato",add=T,border=NA,outline=F)

# y-axis
axis(2,at=seq(0,4,1),lab=seq(0,4,1),pos=0,las=2,lwd=1.5,cex.axis=1.5)

# labs
labx = expression(paste("N=","p(1-p)/",sigma**2,""[gen]))
laby = expression(paste("log"[10],"(N)"))
mtext(laby,2,1,cex=1.5,adj=1)
legend(3,4,legend=c(labx,"Mitot. Segreg."),fill=c("royalblue","tomato"),bty="n",border="white"


# Add boxplot stats to vioplot
```

```
data_tmp=list(bn1.m, bn2.m)

for (i in c(1,2)) {
    y=round(median(unlist(data_tmp[i])),2)
    text(i+0.175,y,lab=round(medians[i],1),col="white",cex=0.7,font=2)
    y=round(summary(unlist(data_tmp[i]))[2],2)
    text(i,y,lab=round(first[i],1),col="white",cex=0.7,font=2)
    y=round(summary(unlist(data_tmp[i]))[5],2)
    text(i,y,lab=round(third[i],1),col="white",cex=0.7,font=2)
}
```

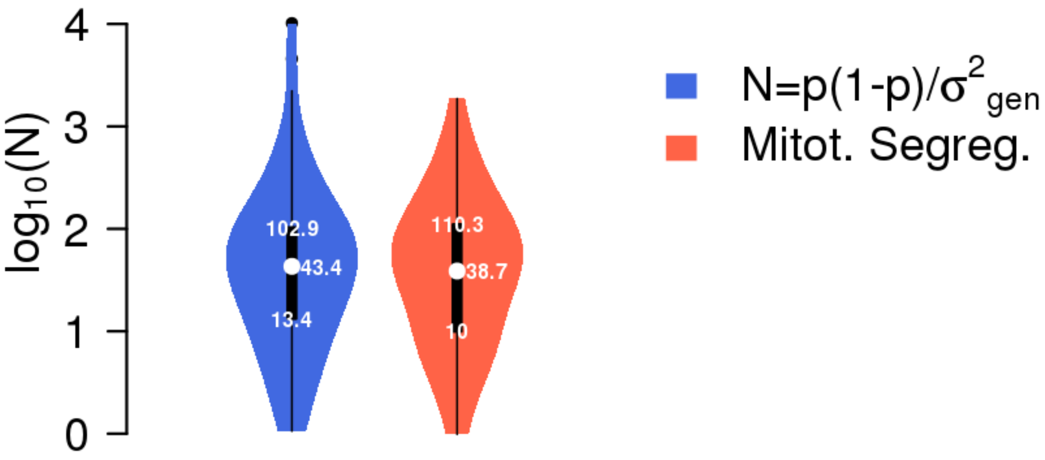

3.6 Plot age correlations (Fig. 2)

In [79]: %%R

```
countHq = function(row){
    cheek = row[1]
    blood = row[2]
    if(cheek>=0.01|blood>=0.01){
        return(1)
    }else{
```

```
            return(0)
        }
    }
```

In [80]: %%R

```
# count the number of heteroplasmic sites with MAF ≥ 1% (in either tissue) per individual
data = quartets

data$Nmother = apply(quartets[,c(5,8)],1,FUN=countHq)
data$Nchild = apply(quartets[,c(11,14)],1,FUN=countHq)
nsites = aggregate(cbind(Nmother,Nchild) ~ family, data=data,FUN=sum)
```

In [81]: %%R

```
head(nsites)
```

```
  family Nmother Nchild
1   M117       0      1
2   M132       2      1
3   M137       3      1
4   M188       0      1
5   M190       3      3
6   M196       1      1
```

In [82]: %%R -i sampAges

```
colnames(sampAges) = c("mother","motherAgeCollection","child","childAgeCollection")
```

In [83]: %%R

```
# age in days
head(sampAges)
```

```
  mother motherAgeCollection  child childAgeCollection
0   M132               16658 M132C1               7460
1   M137               14294 M137C2               6202
2   M186               15938 M186C2               3504
3   M188               17714 M188C2               3451
4   M190               18761 M190C2               5866
5   M195               11826 M195C1               2752
```

In [84]: %%R

```
ageEffect = merge(nsites,sampAges,by.x="family",by.y="mother", all.y=TRUE)

# for samples without heteroplasmic sites, merging produces NAs, so we transformed them to zer
ageEffect[is.na(ageEffect)] = 0
```

In [85]: %%R

```
# the age of the mother at the time of conception of the child is assumed to be
# the current age of the mother, less the current age of the child, less nine months (in days 
ageEffect[["motherAgeFertilization"]] = ageEffect[["motherAgeCollection"]] - (ageEffect[["chil
```

In [86]: %%R -w 4 -h 4 -u in -r 144

```
# colors
black = "black"
m_col = "royalblue1"
c_col = "tomato1"

# transparent colors
mother = rgb(matrix(col2rgb(m_col),1,3),alpha=120,maxColorValue=255)
child  = rgb(matrix(col2rgb(c_col),1,3),alpha=120,maxColorValue=255)
borders = c(m_col,c_col)

# plot margins
par(oma=c(0,0,0,0))
par(mar=c(0.5,2.1,0,0))

# Mother data only
d = ageEffect[,c("Nmother","motherAgeCollection")]
plot(1:10,1:10,xlim=c(15,60),ylim=c(-1.5,5),type="n",frame=F,axes=F,xlab="",ylab="",main="")

points(d[["motherAgeCollection"]]/365,d[["Nmother"]],pch=23,col=borders[1],lwd=1,cex=1,bg=mothe
r3=glm(Nmother~motherAgeCollection,data=d,family="poisson")
p3=round(summary(r3)$coefficients[2,4],3)
fit3=data.frame(age=r3$data$motherAgeCollection/365,f=r3$fitted.values)
fit3=fit3[order(fit3$age),]
lines(fit3$age,fit3$f,col=m_col,lwd=2.5)
x1=min(fit3$age)
x2=max(fit3$age)
pmother=round(summary(r3)$coefficients[2,4],2)

# Child data only
par(new=T)
c = ageEffect[,c("Nchild","motherAgeFertilization")]
points(c[["motherAgeFertilization"]]/365,c[["Nchild"]],pch=21,col=borders[2],bg=child,lwd=1,ce
r3=glm(Nchild~motherAgeFertilization,data=c,family="poisson")
fit3=data.frame(con=r3$data$motherAgeFertilization/365,f=r3$fitted.values)
fit3=fit3[order(fit3$con),]
lines(fit3$con,fit3$f,col=c_col,lwd=2.5)
pchild=round(summary(r3)$coefficients[2,4],3)

# labs
axis(side=1,at=seq(15,60,by=5),lab=NA,lwd=2,pos=-0.25,cex.axis=0.75)
axis(side=2,at=0:5,lwd=2,pos=14.5,las=2,cex.axis=0.75)
mtext("number of point heteroplasmies",2,1.3,at=2.5,cex=0.75,font=2)
mtext("maternal age (years)",1,-0.5,cex=0.75,font=2)
for (i in seq(15,60,by=5)){text(i,-0.75,lab=i,cex=0.75)}

M=paste(paste("mother","p=",sep="  "),pmother,sep="")
C=paste(paste("child","p=",sep="      "),pchild,sep="")
legend(15,4.6,legend=c(M,C),col=borders,
       pt.bg=c(mother,child),pch=c(23,21),pt.cex=1.2,pt.lwd=1.2,
       cex=0.6,title="Poisson model",box.col="gray",box.lwd=1.5,bg=NA)
```

```
x3=min(fit3$con)
x4=max(fit3$con)

lines(c(x1,x2),c(-1,-1),lwd=1.5,col=m_col)
lines(c(x3,x4),c(-1.1,-1.1),lwd=1.5,col=c_col)
mtext("fertilization",1,-1.25,at=27,cex=0.75,col=c_col,font=2)
mtext("collection",1,-1.25,at=45,cex=0.75,col=m_col,font=2)
```

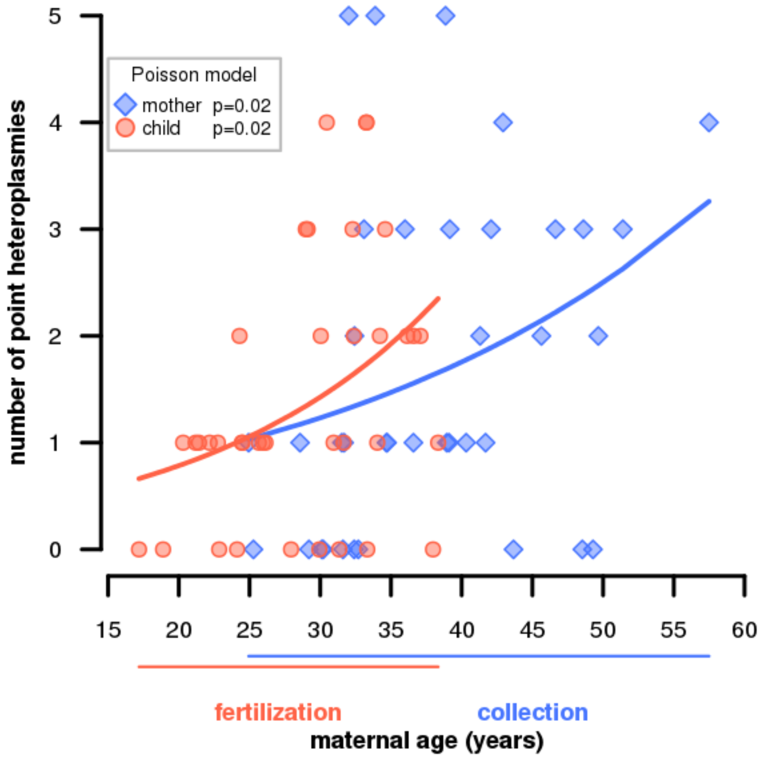


```
In [ ]:
```
